# METTL16 is Required for Meiotic Sex Chromosome Inactivation and DSB Formation and Recombination during Male Meiosis

**DOI:** 10.1002/advs.202406332

**Published:** 2024-11-28

**Authors:** Lisha Yin, Nan Jiang, Wenjing Xiong, Shiyu Yang, Jin Zhang, Mengneng Xiong, Kuan Liu, Yuting Zhang, Xinxin Xiong, Yiqian Gui, Huihui Gao, Tao Li, Yi Li, Xiaoli Wang, Youzhi Zhang, Fengli Wang, Shuiqiao Yuan

**Affiliations:** ^1^ Institute of Reproductive Health Tongji Medical College Huazhong University of Science and Technology Wuhan 430030 China; ^2^ Laboratory of Animal Center Huazhong University of Science and Technology Wuhan 430030 China; ^3^ Department of Obstetrics and Gynecology The Central Hospital of Wuhan Tongji Medical College Huazhong University of Science and Technology Wuhan 430014 China; ^4^ School of Pharmacy Hubei University of Science and Technology Xianning 437100 China

**Keywords:** DSB formation, m^6^A, meiosis, METTL16, MSCI, recombination, translation

## Abstract

Meiosis in males is a critical process that ensures complete spermatogenesis and genetic diversity. However, the key regulators involved in this process and the underlying molecular mechanisms remain unclear. Here, we report an essential role of the m^6^A methyltransferase METTL16 in meiotic sex chromosome inactivation (MSCI), double‐strand break (DSB) formation, homologous recombination and SYCP1 deposition during male meiosis. METTL16 depletion results in a significantly upregulated transcriptome on sex chromosomes in pachytene spermatocytes and leads to reduced DSB formation and recombination, and increased SYCP1 depositioin during the first wave of spermatogenesis. Mechanistically, in pachytene spermatocytes, METTL16 interacts with MDC1/SCML2 to coordinate DNA damage response (DDR) and XY body epigenetic modifications that establish and maintain MSCI, and in early meiotic prophase I, METTL16 regulates DSB formation and recombination by regulating protein levels of meiosis‐related genes. Furthermore, multi‐omics analyses reveal that METTL16 interacts with translational factors and controls m^6^A levels in the RNAs of meiosis‐related genes (e.g., *Ubr2*) to regulate the expression of critical meiotic regulators. Collectively, this study identified METTL16 as a key regulator of male meiosis and demonstrated that it modulates meiosis by interacting with MSCI‐related factors and regulating m^6^A levels and translational efficiency (TE) of meiosis‐related genes.

## Graphical abstract

METTL16 predominantly controls the expression of critical meiotic genes essential for DSB formation and recombination in early meiotic spermatocytes and is involved in the expansion of DNA damage response (DDR) factors and epigenetic modifications in the XY body for the establishment and maintenance of meiotic sex chromosome inactivation (MSCI) in pachytene spermatocytes, thus ensuring meiotic progression.

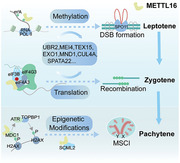

## 1 Introduction

Meiosis, a conserved cell division process, involves two chromosomal segregations after DNA replication to generate haploid germ cells for reproduction. Meiotic prophase I is a relatively long and complicated process of ordered events, such as DNA double‐strand break (DSB) formation, recombination, synapsis, and desynapsis. Abnormalities in the meiotic process can lead to infertility, aneuploidy‐related birth defects, and pregnancy failure in mammals, indicating that fidelity to this process is paramount for preserving fertility and genetic inheritance.

At the onset of male meiosis, cohesins and HORMAD1 coordinate synaptonemal complex (SC) formation, while the pre‐DSB machinery, including IHO1,^[^ ^1^ ^]^ MEI4,^[^ ^2^ ^]^ REC114,^[^ ^2^, ^3^ ^]^ ANKRD31,^[^ ^3^, ^4^ ^]^ and MEI1,^[^ ^5^, ^6^ ^]^ are recruited to the axes to provide a suitable molecular environment for SPO11‐mediated DNA breakage.^[^ ^7^, ^8^ ^]^ The SC is critical for proper homologous synapses, consisting of transverse filaments (SYCP1), lateral elements (SYCP3 and SYCP2), and central elements (SYCE3, SYCE1, SIX6OS1, SYCE2, and TEX12).^[^ ^9^, ^10^ ^]^ In pachytene spermatocytes, homologous chromosomes complete synapsis with limited synaptic events occurring in the pseudoautosomal regions (PAR) of sex chromosomes. Notably, meiotic sex chromosome inactivation (MSCI), a characteristic of meiotic silencing of unsynapsed chromatin (MSUC) in males, occurs at the pachytene stage. MSCI is a critical checkpoint during meiotic prophase I; those with failed MSCI undergo spermatocyte elimination in the mid‐pachytene stage.^[^ ^11^, ^12^ ^]^ During MSCI initiation, DNA‐damage response (DDR) factors, including ATR and TOPBP1, are first recruited and spread along the chromosome axes in BRCA1‐dependent pathway, while their subsequent expansion to the chromosome‐wide domain is dependent on γH2AX‐MDC1.^[^ ^12^ ^]^ Although several recognized downstream pathways of γH2AX‐MDC1 have been recognized to play roles in MSCI and XY body formation, including the SETDB1‐HP1,^[^ ^13^ ^]^ Fanconi Anaemia (FA),^[^ ^14^ ^]^ RNF8‐related ubiquitination,^[^ ^15^, ^16^ ^]^ SCML2‐USP7,^[^ ^15^, ^17^, ^18^ ^]^ PARP2,^[^ ^19^ ^]^ and SUMO^[^ ^20^, ^21^ ^]^ pathways, the precise control of MSCI during meiotic prophase I by genetic regulation network remains poorly understood.

*N6*‐methyladenosine (m^6^A) is the most common RNA modification involved in mRNA splicing, export, degradation, stability, and translation.^[^ ^22^ ^]^ METTL16, a newly identified m^6^A writer, was the second m^6^A methyltransferase identified. In contrast to the classical methyltransferase METTL3‐METTL14 complex, which catalyzes the m^6^A modification of thousands of transcripts, METTL16 modifies distinct substrates, such as snRNAs, XIST, and MAT2A transcripts.^[^ ^23^, ^24^, ^25^, ^26^ ^]^ A recent study verified the role of METTL16 in leukemogenesis and leukemia stem cell self‐renewal in the m^6^A‐dependent pathway.^[^ ^27^ ^]^ However, two other studies revealed that METTL16 can regulate several processes through the m^6^A‐independent pathway and cooperate with translation‐related proteins to promote tumorigenesis.^[^ ^28^, ^29^ ^]^ In addition, METTL16 is involved in regulating mouse embryonic development,^[^ ^30^ ^]^ and in *Ddx4‐Cre* induced germ cell‐specific knockout male mice, in which deletion occurs at embryonic day 15.5, the mice displayed a Sertoli cell‐only phenotype;^[^ ^24^ ^]^ however, whether METTL16 functions in the male meiotic process is unknown.

Here, we identified a novel role of the m^6^A methyltransferase METTL16 in the meiotic process. Using a *Ddx4‐Cre* ^ERT2^‐mediated tamoxifen‐inducible knockout approach, we demonstrated that METTL16 controls the establishment and maintenance of MSCI during both first wave and steady‐state spermatogenesis since METTL16‐deficient pachytene spermatocytes exhibited failed spreading of DDR factors among chromosome‐wide domains and abnormal epigenetic modifications. Notably, we also discovered that meiocytes with METTL16 deletion displayed reduced DSB formation and recombination and abnormal SYCP1 deposition in early meiotic prophase I specifically during the first wave of spermatogenesis. Mechanistically, we found that METTL16 could interact with MDC1/SCML2 to coordinate proper DDR pathway and epigenetic modifications of the XY body. Importantly, further RIP‐seq, MeRIP/m^6^A‐seq, Ribo‐seq, and IP‐MS analyses revealed that METTL16 interacts with translational factors and mediates m^6^A levels in meiosis‐related genes (e.g., *Ubr2*). This study identified METTL16 as a multifunctional regulator that ensures meiotic progression by cooperating with MSCI‐related and translation‐related proteins and regulating m^6^A levels in meiosis‐related genes during spermatogenesis.

## 2 Results

### 2.1 METTL16 is Essential for Male Meiosis

**Figure 1:**
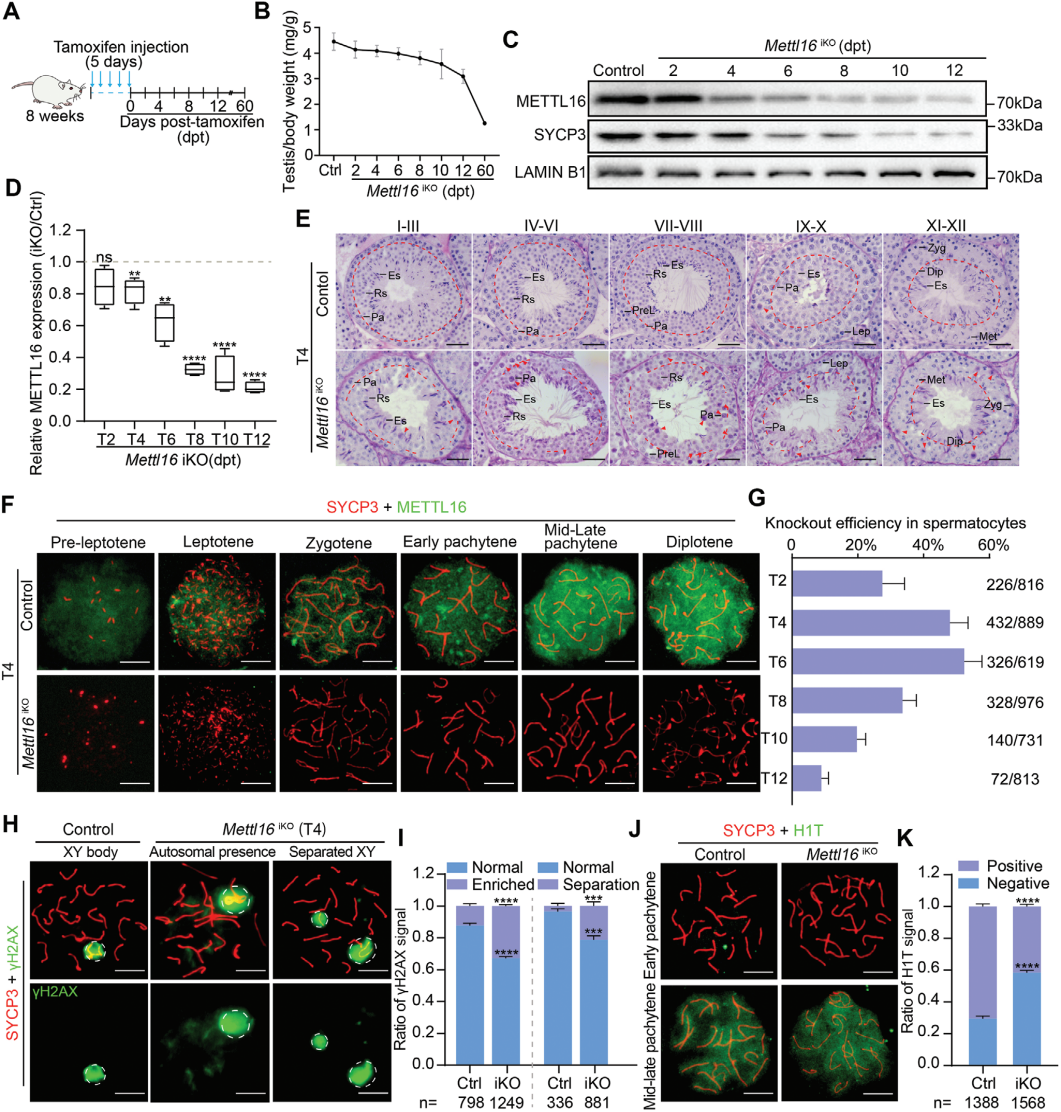
METTL16 is essential for meiosis progression during spermatogenesis. A) A regimen of tamoxifen treatment in 8‐week‐old *Mettl16^flox/−^Ddx4‐Cre* ^ERT2 ^mice is shown. Testes were harvested at 2, 4, 6, 8, 10, 12, and 60 days post‐tamoxifen (dpt) for investigation. B) Ratios of testis/body weight of Control (Ctrl) and *Mettl16^flox/−^Ddx4‐Cre* ^ERT2 ^(*Mettl16* ^iKO^) adult mice at 2, 4, 6, 8, 10, 12, and 60 dpt are shown, respectively (n = 4 for each group). C) Western blot analysis of METTL16 and SYCP3 in Control and *Mettl16* ^iKO^ adult mouse testes at 2, 4, 6, 8, 10, and 12 dpt. LAMIN B1 was used as a loading control. D) Quantification of METTL16 protein level in (C). Data were presented as mean ± SEM, n = 3. ns, not significant. **P < 0.01, ****P < 0.0001. E) Histological analyses of different stages of testicular sections from Control and *Mettl16* ^iKO^ adult mice at 4 dpt (T4) are shown. Twelve stages were divided into 5 groups: stage I‐III, stage IV‐VI, stage VII‐VIII, stage IX‐X, and stage XI‐XII. Abbreviations: PreL, preleptotene; Lep, leptotene; Zyg, zygotene; Pa, pachytene; Dip, diplotene; Met, metaphase; Rs, round spermatid; Es, elongating spermatid. Red arrowheads indicate apoptotic cells. The dashed red line indicates the layer of pachytene/diplotene spermatocytes. Scale bars = 50 µm. F) Expression pattern of METTL16 in spermatocytes and knockout validation using surface nuclear spread analysis of Control and *Mettl16* ^iKO^ mouse spermatocytes at T4. Scale bars = 10 µm. G) Knockout efficiency in *Mettl16* ^iKO^ mouse spermatocytes at different time points. The knockout efficiency was measured based on chromosome spread assay co‐staining with SYCP3/METTL16. H) Nuclear spread analysis and quantification of γH2AX in pachytene spermatocytes from Control and *Mettl16* ^iKO^ mice at T4 are shown. Scale bars = 10 µm. The dashed white lines demarcate sex chromosomes (XY body). The abbreviation of left panel in quantification part: Normal, no signal in autosomes; Enriched, signal in autosomes. The abbreviation of right panel in quantification part: Normal, no XY separation; Separation, XY separation. I) The quantified data were presented as mean ± SEM for H). The spermatocytes were counted from three control and *Mettl16* ^iKO^ mice (T4), respectively. ***P < 0.001, ****P < 0.0001. J) Nuclear spread analysis and quantification of H1T in pachytene spermatocytes from Control and *Mettl16* ^iKO^ mice at T4 are shown. Scale bars = 10 µm. K) The quantified data were presented as mean ± SEM. The spermatocytes were counted from three control and *Mettl16* ^iKO^ mice, respectively. ****P < 0.0001. The abbreviation in quantification part: Positive: H1T‐positive mid‐late pachytene spermatocytes; Negative: H1T‐negative early pachytene spermatocytes.

To determine the function of METTL16 in spermatogenesis, we first examined the expression pattern of METTL16 in purified testicular cells using qPCR and western blot assays. The results showed that METTL16 is expressed in spermatogonia, spermatocytes, round spermatids, and Sertoli cells, with a preferential localization in the cytoplasm (Figure S1A–D, Supporting Information). We then generated a germ cell‐specific *Mettl16* knockout mouse model (*Mettl16^flox/−^* *Ddx4‐Cre*, here called *Ddx4‐*Cre‐cKO) by crossing the consecutively expressed *Ddx4*‐Cre transgenic mice with *Mettl16* ^flox/flox^ mice and found that the *Ddx4‐*Cre‐cKO males exhibited complete germ cell loss and Sertoli cell‐only phenotype from postnatal day 12 (P12) (Figure S1E,F, Supporting Information). By staining for the germ cell marker DDX4 and the spermatogonial marker PLZF using P3, P5 and P7 testis sections, we found that the number of DDX4‐positive cells was reduced at P7, and the number of PLZF‐positive cells was increased at P5 and P7 in *Ddx4‐*Cre‐cKO testes, suggesting that METTL16 is essential for spermatogonial differentiation and germ cell survival (Figure S1G–I, Supporting Information). To circumvent the pre‐meiotic germ cell loss in *Ddx4‐*Cre‐cKO males, we generated *Mettl16^flox/−^* *Ddx4*‐*Cre* ^ERT2^ mice (referred to as *Mettl16* ^iKO^ or iKO) for tamoxifen‐induced inactivation of *Mettl16* specifically in germ cells (**Figure** **1**A). Intraperitoneal tamoxifen injection in *Mettl16^flox/−^* *Ddx4*‐*Cre* ^ERT2^ adult males resulted in a progressive decrease in testis/body weight and protein levels of METTL16 and SYCP3 (marker for spermatocytes) over time (Figure 1B–D). Histological analyses of *Mettl16* ^iKO^ adult males at different days post‐tamoxifen treatment (T4, T6, T8, or T10) showed varying degrees of defects in spermatogenesis, particularly pachytene or diplotene spermatocyte loss and apoptotic cell appearance (Figure 1E; Figure S2A, Supporting Information). At T6 and later time points, spermatocyte loss was very severe in many tubules, with obvious loss of mid‐pachytene at stages IV‐VIII and almost no late pachytene or diplotene at stages IX‐XII (Figure S2A, Supporting Information). We, therefore, selected T4 testes for further study. At T4, we quantified the number of spermatocytes stage by stage in the seminiferous tubules and found that the number of spermatocytes was also significantly reduced in *Mettl16* ^iKO^ mice at all stages compared to controls (Figure 1E; Figure S2B, Supporting Information). In addition, the testes of T60 *Mettl16* ^iKO^ mice had many Sertoli cell‐only tubules, and the epididymis was almost empty (Figure S2C–E, Supporting Information). Consistent with histological result, the testes of T60 *Mettl16* ^iKO^ mice were smaller than those of control mice (Figure S2F, Supporting Information). Taken together, these results indicate an essential role of METTL16 in male meiosis.

To confirm the role of METTL16 in meiosis, we performed a chromosome spread analysis of T4 *Mettl16* ^iKO^ mice. Cellular localization of METTL16 in spermatocytes was assessed using immunofluorescence (IF) on chromosome‐spread slides. In control mice, METTL16 was expressed from preleptotene to diplotene during prophase I of meiosis and peaked in pachytene and diplotene spermatocytes. In contrast, METTL16 signal was not detectable in some spermatocytes of *Mettl16* ^iKO^ mice (Figure 1F), indicating that METTL16 depletion in meiotic cells was effective. The knockout efficiency of *Mettl16* in spermatocytes at different time points after tamoxifen injection varied and peaked at T4 and T6 at ≈50% (Figure 1G; Figure S2G, Supporting Information). We then examined meiotic progress in the control and *Mettl16* ^iKO^ mice at T4 by staining for different meiosis‐indicative markers. γH2AX is a marker for DSB formation at early meiocytes and reflects DSB repair indirectly at pachytene/diplotene spermatocytes. Compared to controls, the γH2AX signal showed abnormal localization in *Mettl16* ^iKO^ pachytene spermatocytes (Figure 1H). Almost all pachytene spermatocytes of control mice displayed γH2AX signal accumulation in the XY body (Figure 1H). In contrast, in *Mettl16* ^iKO^ mice, the percentage of autosomal presence (≈32.7% in iKO vs. ≈12.2% in Ctrl) and separated XY signals (≈21.3% in iKO vs. ≈3.4% in Ctrl) were significantly elevated (Figure 1I). Considering that severe pachytene loss occurs at stage IV‐VI and beyond, indicating mid‐late pachytene loss, we co‐immunostained histone H1T with SYCP3 to examine the proportion of early and mid‐late pachytene spermatocytes. Compared with control mice, the percentage of H1T‐negative early pachytene was significantly increased (≈58.3% in iKO vs. ≈29.6% in Ctrl) in *Mettl16* ^iKO^ mice, indicating that the transition from early pachytene to mid‐pachytene was affected by METTL16 deletion (Figure 1J,K). Together, these results demonstrate that METTL16 is essential for the progression of pachytene spermatocytes.

### 2.2 METTL16 is Required for the Amplification of DDR Factors to Facilitate MSCI Establishment and Maintenance

**Figure 2:**
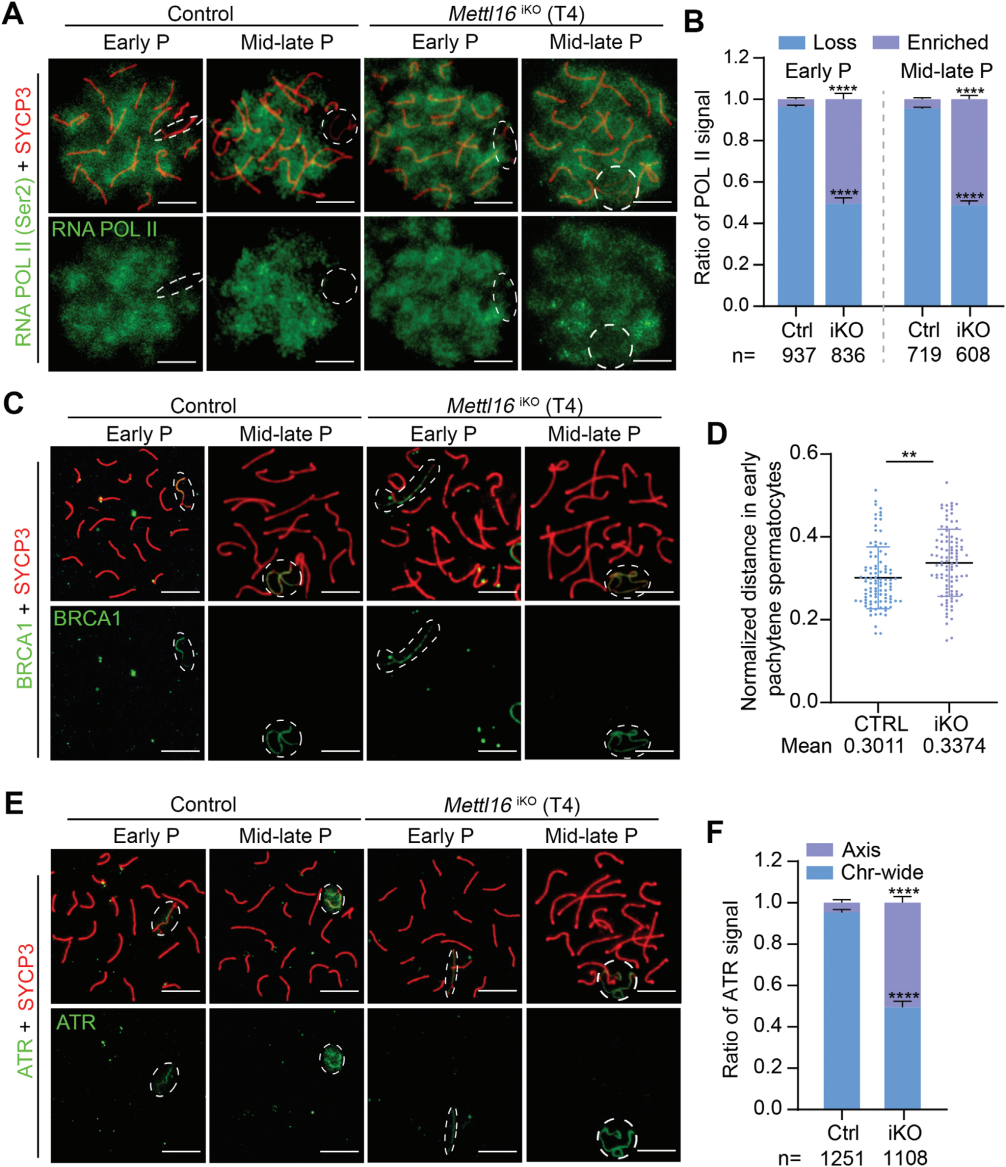
Ablation of METTL16 leads to failed amplification of DDR factors and MSCI establishment and maintenance. A) Representative images of nuclear spread analysis of RNA POL II in early and mid‐late pachytene spermatocytes from Control (Ctrl) and *Mettl16* ^iKO^ (iKO) mice at T4 are shown. Scale bars = 10 µm. Abbreviation: Early P, early pachytene; Mid‐late P, mid‐late pachytene. B) The quantification of RNA POL II signal for (A). Abbreviation: Loss, no signal in XY body; Enriched, signal in XY body. The indicated number of spermatocytes counted from three control and *Mettl16* ^iKO^ mice are shown at the bottom of the histogram, respectively. The quantified data were presented as mean ± SEM. ****P < 0.0001. n = 3 mice. C,D) Nuclear spread analysis C) and distance quantification of BRCA1 D) in pachytene spermatocytes from Control and *Mettl16* ^iKO^ mice at T4 are shown. Abbreviation: Early P, early pachytene; Mid‐late P, mid‐late pachytene. The normalized distance is the linear distance between two distal ends of XY axes normalized to the nuclear diameter in early pachytene spermatocytes. The quantified data were presented as mean ± SEM. **P < 0.01. n = 3 mice. Scale bars = 10 µm. E,F) Representative images of nuclear spread analysis E) and quantification F) of ATR in early and mid‐late pachytene spermatocytes from Control and *Mettl16* ^iKO^ mice at T4 are shown. The quantification was different types of ATR signals in mid‐late pachytene spermatocytes. Abbreviation: Axis, ATR signal only on axis; Chr‐wide, ATR signal on chromosome‐wide domain. The quantified data were presented as mean ± SEM. The indicated number of spermatocytes were counted from three Ctrl and iKO mice, respectively. ****P < 0.0001. Scale bars = 10 µm.

Considering that defective MSCI could lead to loss of mid‐pachytene and impaired transition from early to mid‐late pachytene,^[^ ^31^ ^]^ we investigated whether the MSCI is disrupted in *Mettl16^iKO^* pachytene spermatocytes. To this end, RNA polymerase II (POL II) was used to detect transcription levels in the sex chromosomes. Unlike the exclusion from sex chromosomes in control pachytene spermatocytes, in *Mettl16* ^iKO^ pachytene spermatocytes, POL II (Ser2) signals were observed among sex chromosomes, whether in early or mid‐late pachytene (Early P: ≈50.5% in iKO vs. 3.7% in Ctrl; Mid‐late P:∼≈51.0% in iKO vs. 4.5% in Ctrl) (**Figure** **2**A,B; Figure S3A, Supporting Information). To confirm this phenotype, another POL II (Ser5) antibody was also used to determine the transcriptional status of the sex chromosomes of pachytene spermatocytes in control and *Mettl16* ^iKO^ mice. An abnormal enrichment of POL II signal on sex chromosomes was also observed in *Mettl16* ^iKO^ pachytene spermatocytes (Figure S3B, Supporting Information). Furthermore, since a previous study reported that impaired MSCI was always accompanied by trapped γH2AX on autosomes,^[^ ^32^ ^]^ we co‐stained POL II with SYCP3 and γH2AX and found that the trapped γH2AX signals on autosomes were detected in MSCI‐defective pachytene spermatocytes of *Mettl16* ^iKO^ mice (Figure S3C, Supporting Information). These results indicate that MSCI was disrupted in *Mettl16* ^iKO^ mice. To investigate whether homologous recombination was impaired in *Mettl16* ^iKO^ pachytene spermatocytes, the recombination proteins RPA2 and RAD51 were quantified, and no obvious abnormality was observed (Figure S3D–I, Supporting Information), indicating that homologous recombination was not impaired in pachytene spermatocytes of adult *Mettl16* ^iKO^ mice.

Since the DDR pathway is essential for establishing and maintaining MSCI, we measured several important DDR factors in *Mettl16* ^iKO^ mice. BRCA1, a meiotic silencing sensor, recruits DDR factors to unsynapsed chromosome axes. Compared to control mice, no obvious abnormalities in BRCA1 localization were observed in *Mettl16* ^iKO^ mice, indicating that the recruitment of DDR factors to the unsynapsed axes was not affected in METTL16‐deficient spermatocytes (Figure 2C). To determine the chromatin conformation of sex chromosomes, we quantified the distance between the ends of the XY axes in early pachytene spermatocytes based on BRCA1 staining. We found XY axes in *Mettl16* ^iKO^ mice were longer than in control mice (Figure 2D), suggesting that METTL16 is involved in sex chromosome conformation during the early pachytene stage. HORMAD1 is localized at unsynapsed chromosomes, and is essential for the recruitment of BRCA1.^[^ ^33^ ^]^ The signal of HORMAD1 was always on the unsynapsed sex chromosomes, and no obvious defects were observed in *Mettl16* ^iKO^ mice (Figure S4A, Supporting Information). ATR and TOPBP1 are classical DDR proteins that show axis localization during early pachytene and chromosome‐wide localization during mid‐late pachytene in XY body. We found that the ratio of mid‐late pachytene spermatocytes with failed expansion of ATR factor to chromosome‐wide domain was significantly elevated in *Mettl16* ^iKO^ mice, compared with that of controls (≈50.5% in iKO vs. ≈4.6% in Ctrl), while the ratio of that with chromosome‐wide signal was significantly reduced (≈49.5% in iKO vs. ≈95.4% in Ctrl) (Figure 2E,F). Similarly, the expansion of TOPBP1 and p‐ATR (phospho‐S428 ATR, an active form of ATR) to chromosome‐wide domain of sex chromosomes were both failed in mid‐late pachytene spermatocytes in *Mettl16* ^iKO^ mice compared to the controls (Figure S4B,C, Supporting Information). Furthermore, the signal for ATRIP, an ATR‐interacting protein usually located on the unsynapsed XY axes, exhibited a higher rate of complete absence on sex chromosomes in *Mettl16* ^iKO^ mice (Figure S4D, Supporting Information). Since METTL16 regulates MRN (MRE11‐RAD50‐NBS1) complex‐mediated DNA end resection and homologous recombination in somatic cells,^[^ ^25^ ^]^ we examined the localization of MRN in pachytene spermatocytes from control and *Mettl16* ^iKO^ mice. Like the control, these three proteins all exhibited persistent accumulation on the sex chromosomes in *Mettl16* ^iKO^ mice, demonstrating that METTL16 depletion does not influence the enrichment of the MRN complex on the sex chromosomes (Figure S4E–G, Supporting Information). Collectively, these data suggest that METTL16 is involved in the spreading of DDR proteins to the entire XY body for MSCI establishment and maintenance.

### 2.3 METTL16 Modulates the Proper Epigenetic Modifications on Sex Chromosomes

**Figure 3:**
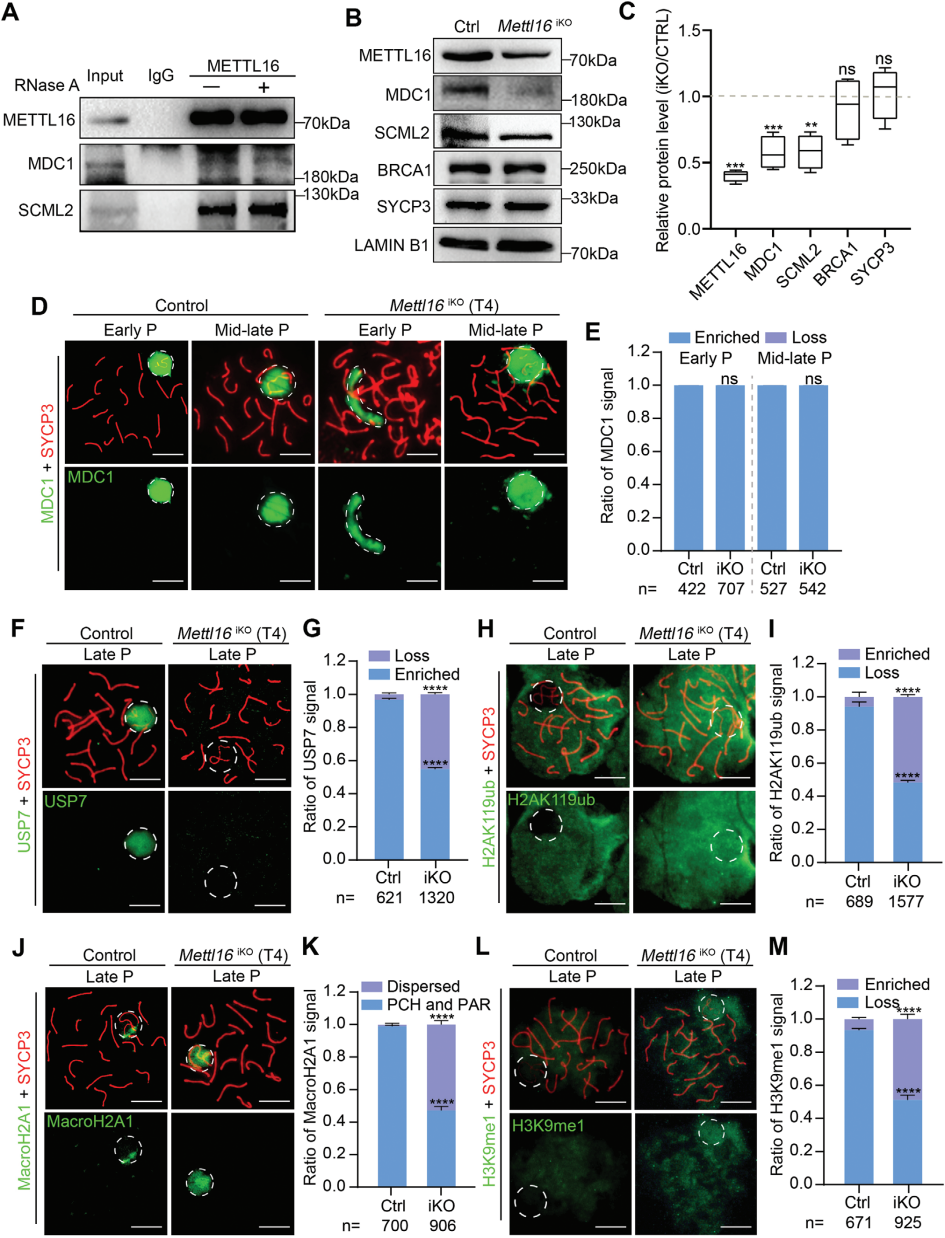
METTL16 interacts with MDC1/SCML2 and is required for epigenetic programming of male meiotic sex chromosomes. A) Co‐IP assay for the confirmation of the interaction between METTL16 and MDC1/SCML2 in pachytene spermatocytes. B,C) Western blot and quantification of MDC1, SCML2, BRCA1 and SYCP3 in Ctrl and iKO pachytene spermatocytes. LAMIN B1 represents total protein and was used as normalization. The quantified data were presented as mean ± SEM. **P < 0.01, ***P < 0.001. ns, not significant. n = 8 mice. D,E) Representative images of nuclear spread analysis D) and quantification E) of MDC1 in pachytene spermatocytes from Control and *Mettl16* ^iKO^ mice (T4) are shown. Abbreviation: Early P, early pachytene; Mid‐late P, mid‐late pachytene. The abbreviation in quantification part: Loss, no signal in XY body; Enriched, signal in XY body. Scale bars = 10 µm. F–M) Representative images of nuclear spread analysis and quantification of USP7 F,G), H2AK119ub H,I), MacroH2A1 J,K), and H3K9me1 L,M) in late pachytene spermatocytes from Control and *Mettl16* ^iKO^ mice (T4) are shown. Abbreviation: Late P, late pachytene. The abbreviation in quantification part: Loss, no signal in XY body; Enriched, signal in XY body; Dispersed, dispersed signal in XY body; PCH and PAR, signal in PCH and PAR region. The quantified data were presented as mean ± SEM. The indicated number of late pachytene spermatocytes were counted from three Ctrl and iKO mice, respectively. ****P < 0.0001. Scale bars = 10 µm.

To further investigate the molecular network by which METTL16 regulates MSCI on sex chromosomes, we performed immunoprecipitation mass spectrometry (IP‐MS) using a METTL16 antibody to identify its interacting proteins in spermatocytes and found that METTL16 could potentially interact with MDC1 and SCML2 (Table S1, Supporting Information). Further Co‐immunoprecipitation (Co‐IP) experiments demonstrated the interaction of METTL16 with MDC1 and SCML2 in a RNase‐independent manner (**Figure** **3**A). Since MDC1 is critical for the amplification of DDR factors from the axis to the chromosome‐wide domain^[^ ^34^ ^]^ and SCML2 is a polycomb protein that regulates essential epigenetic and post‐translational modifications of XY chromosomes,^[^ ^15^, ^17^, ^18^ ^]^ we performed western blot assays on isolated pachytene spermatocytes from control and *Mettl16^iKO^* mice and found reduced protein levels of both MDC1 and SCML2 in *Mettl16^iKO^* mice (Figure 3B,C). We then examined the localization of MDC1 using chromosome spread slides, and found that the enrichment of MDC1 on sex chromosomes was not altered in *Mettl16^iKO^* mice compared to control mice (Figure 3D,E). Since the protein level of SCML2 was reduced in *Mettl16* ^iKO^ pachytene spermatocytes, and there was an interaction between SCML2 and USP7, which is involved in the deubiquitination of H2A in somatic cells^[^ ^17^, ^35^ ^]^ and accumulates on sex chromosomes in an SCML2‐dependent manner,^[^ ^17^ ^]^ we next detected the localization of USP7 on sex chromosomes of late pachytene spermatocytes in control and *Mettl16* ^iKO^ mice. The results showed that the proportion of late pachytene in the absence of USP7 was significantly higher in *Mettl16* ^iKO^ mice than in control mice (≈45.2% in iKO vs. ≈3.3% in Ctrl) (Figure 3F,G). Because the deubiquitinase is involved in controlling the exclusion of H2AK119ub from the sex chromosomes in late pachytene spermatocytes,^[^ ^17^ ^]^ we detected the localization of H2AK119ub. As expected, the exclusion of H2AK119ub was impaired in ≈51.8% of late pachytene spermatocytes in *Mettl16* ^iKO^ mice, while in control, this ratio was ≈5.9% (Figure 3H,I). In addition, since the localization of histone variant MacroH2A1 that accumulates locally at unsynapsed axes in early pachytene spermatocytes and at pericentric heterochromatin (PCH) and PAR in mid‐late pachytene spermatocytes was relied on H2AK119ub,^[^ ^17^, ^36^, ^37^ ^]^ we also detected the localization of MacroH2A1. Interestingly, we found an increase of MacroH2A1 with dispersed signal in late pachytene spermatocytes in *Mettl16* ^iKO^ mice compared with controls (≈52.8% in iKO vs. ≈1.0% in Ctrl) (Figure 3J,K). Other than MacroH2A1, the localization of H3K9me1 was also closely related to H2AK119ub, which could exclude H3K9me1 from sex chromosomes in late pachytene spermatocytes. In *Mettl16* ^iKO^ mice, H3K9me1 was retained on sex chromosomes in ≈48.9% of late pachytene spermatocytes, compared to ≈6.6% in controls (Figure 3L,M). These results were all consistent with *Scml2* mutant spermatocytes.^[^ ^17^ ^]^ Taken together, METTL16 may interact with SCML2 to regulate its downstream epigenetic modifications.

Since SUMOylation and some other histone modifications are essential epigenetic and post‐translational modifications of XY chromosomes for establishing and maintaining MSCI, we speculated that these might also be affected in METTL16‐deficient pachytene spermatocytes due to a dysregulated DDR pathway. To test this hypothesis, we first examined the localization of small ubiquitin‐related modifier 1 (SUMO‐1)^[^ ^20^, ^21^ ^]^ in pachytene spermatocytes. In control mice, SUMO‐1 was evenly localized on the XY bodies in mid‐late pachytene spermatocytes; however, in ≈51.2% of late pachytene spermatocytes from *Mettl16* ^iKO^ mice, the SUMO‐1 signal disappeared, compared to ≈4.5% in controls (Figure S5A,B, Supporting Information), indicating METTL16‐deficiency could disrupt SUMOylation on sex chromosomes in late pachytene spermatocytes. We then detected the expression of H3K9AC, which is associated with active transcription, and found that H3K9AC was excluded from the sex chromosomes in mid‐late pachytene spermatocytes in control mice, whereas it was highly enriched on the sex chromosomes in *Mettl16* ^iKO^ mice (≈53.0% in iKO vs. ≈5.1% in Ctrl) (Figure S5C,D, Supporting Information). As the SETDB1‐H3K9me3 pathway is essential for MSCI establishment, we examined its expression in control and *Mettl16* ^iKO^ pachytene spermatocytes. Interestingly, we did not observe any significant changes in the expression pattern of both SETDB1 and H3K9me3 in *Mettl16* ^iKO^ pachytene spermatocytes (early to mid‐late) compared to controls (Figure S5E,F, Supporting Information). In addition, the gene repressive mark H3K27me3 was always excluded from the sex chromosomes in late pachytene spermatocytes in controls, and this exclusion was also present in *Mettl16* ^iKO^ mice (Figure S5G, Supporting Information). Since METTL16 could affect FA protein in somatic cells,^[^ ^38^ ^]^ and FA protein is involved in regulating H3K9me2 on sex chromosomes,^[^ ^39^ ^]^ we further detected H3K9me2 modification on the sex chromosomes of pachytene spermatocytes and also found no significant difference between the control and *Mettl16* ^iKO^ mice (Figure S5H, Supporting Information). Other than these, CHD4 is a chromatin remodeler and is enriched on sex chromosomes in mid‐late pachytene spermatocytes, and this enrichment was not altered by METTL16 deletion (Figure S5I, Supporting Information). Collectively, these results indicate that METTL16 is involved in epigenetic modifications, such as SUMOylation and H3K9AC of sex chromosomes of meiotic pachytene spermatocytes, to establish and maintain MSCI.

### 2.4 METTL16‐Deficiency causes Failure of XY‐Linked Gene Silencing in Pachytene Spermatocytes

**Figure 4:**
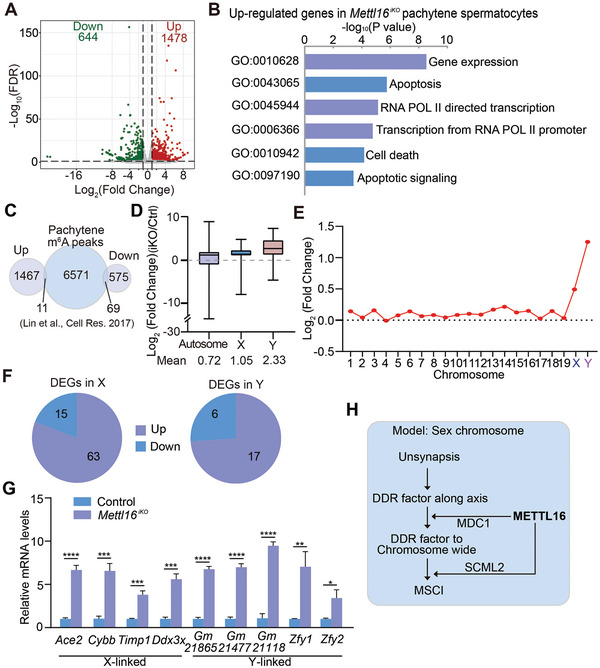
METTL16 deficiency causes failure of XY‐linked gene silencing in pachytene spermatocytes. A) Volcano plot of differentially expressed genes (DEGs) in isolated pachytene spermatocytes from Ctrl and iKO mice at T4 are shown. Cut‐off: log2(Fold Change) ≥ 1.0 or ≤ −1.0, *p*‐value < 0.05. n = 3 mice. B) GO analysis of upregulated genes in *Mettl16* ^iKO^ pachytene spermatocytes (T4). C) Overlap of up‐regulated DEGs and down‐regulated DEGs with published m^6^A peak analysis of pachytene spermatocytes.^[^ ^40^ ^]^ D) Average log2(Fold Change) of differentially expressed genes on autosomes, X, and Y chromosome. E) The mean of log2(Fold Change) of expression levels of all genes on each chromosome. F) Analysis of up‐regulated or down‐regulated DEGs on X and Y chromosomes. G) mRNA quantification of X‐linked and Y‐linked genes in pachytene spermatocytes from Control and *Mettl16* ^iKO^ mice at T4. Data were presented as mean ± SEM. *P < 0.05, **P < 0.01, ***P < 0.001, ****P < 0.0001. n = 5 mice. H) The model of the regulation of METTL16 on sex chromosomes of pachytene spermatocytes in male mice.

To confirm the role of METTL16 in MSCI and determine the consequences of MSCI failure in *Mettl16* ^iKO^ mice, we compared the transcriptome levels in control and *Mettl16* ^iKO^ pachytene spermatocytes. To this end, we isolated pachytene spermatocytes from adult control and *Mettl16* ^iKO^ mice at T4 for RNA‐sequencing (RNA‐Seq) analyses. Pachytene spermatocytes with > 80% purity were used for sequencing and subsequent assays (Figure S6A, Supporting Information). PCA confirmed high repeatability between samples within each group (Figure S6B, Supporting Information). With the cutoff of log2(Fold Change) ≥1.0 or ≤ −1.0, a total of 2122 differentially expressed genes (DEGs) were identified between control and *Mettl16* ^iKO^ pachytene spermatocytes, including 644 downregulated genes and 1478 upregulated genes in *Mettl16* ^iKO^ pachytene spermatocytes relative to controls (**Figure** **4**A, and Table S2, Supporting Information). The GO terms of up‐regulated genes in *Mettl16* ^iKO^ pachytene spermatocytes were closely related to gene expression, transcription, and apoptosis (Figure 4B). When comparing the upregulated and downregulated genes with published m^6^A peak data in pachytene spermatocytes,^[^ ^40^ ^]^ 11 upregulated genes with m^6^A peaks and 69 downregulated genes with m^6^A peaks were identified (Figure 4C). This bioinformatic analysis indicated a weak relationship between METTL16‐directed m^6^A‐modification and gene transcription changes in pachytene spermatocytes.

Given that METTL16‐deficient pachytene spermatocytes failed MSCI, comparison of mRNA levels between autosomes, X chromosome, and Y chromosome was performed. The average fold changes in DEGs at the XY chromosomes, particularly for the Y chromosome (Mean: 2.33), were more significant than those on the autosomes (Mean: 0.72) (Figure 4D). Furthermore, measuring the average transcription levels of all genes on each chromosome also revealed a higher level of gene expression on sex chromosomes in *Mettl16* ^iKO^ pachytene spermatocytes (Figure 4E), suggesting that transcriptomes of the XY chromosomes were more susceptible to METTL16 depletion in pachytene spermatocytes. We focused on DEGs of XY chromosomes (referred to as X/YDEGs) for further analyses. A total of 78 XDEGs and 23 YDEGs were identified, of which 63 XDEGs (≈81%) and 17 YDEGs (≈74%) were upregulated (Figure 4F), suggesting that XY‐linked genes were not repressed in METTL16‐deficient pachytene spermatocytes. To verify these results, we selected several X‐linked (*Ace2*, *Cybb*, *Timp1*, and *Ddx3x*) and Y‐linked (*Gm21865*, *Gm21477*, *Gm21118*, *Zfy1*, and *Zfy2*) genes for quantitative analysis via qPCR. As expected, all selected X‐ and Y‐linked genes were upregulated in *Mettl16* ^iKO^ pachytene spermatocytes compared with the controls (Figure 4G). These results suggest that METTL16 deficiency results in the failure of XY‐linked gene silencing in pachytene spermatocytes and further verifies the indispensable role of *Mettl16* in MSCI establishment and maintenance in male pachytene spermatocytes. Altogether, these data have led to a model for the regulation of METTL16 in MSCI establishment and maintenance in pachytene spermatocytes: METTL16 interacts with MDC1 to regulate the expansion of DDR factors to the chromosome‐wide domain and subsequent epigenetic programming, and it also interacts with SCML2 to control its subsequent modification on the sex chromosomes. These two pathways work together to regulate MSCI establishment and maintenance (Figure 4H).

### 2.5 METTL16 Regulates DSB Formation of Meiocytes of the First Wave of Spermatogenesis

**Figure 5:**
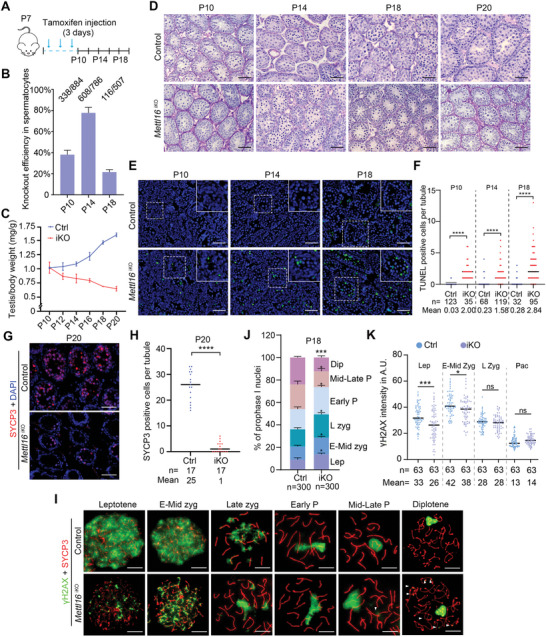
METTL16 regulates DSB formation of spermatocytes of the first wave of spermatogenesis. A) Regimen of tamoxifen treatment in *Mettl16^flox/−^Ddx4‐Cre* ^ERT2^ mice at P7. Testes were harvested at P10, P12, P14, P16, P18, and P20 for investigation. B) Knockout efficiency of spermatocytes in *Mettl16* ^iKO^ mice at different time points. C) Ratio of testis weight/body weight of male Control (Ctrl) and *Mettl16* ^iKO^ (iKO) mice at P10, P12, P14, P16, P18, and P20. n = 3 mice. D) Histological analysis of testes from Control and *Mettl16* ^iKO^ juvenile male mice at different time points. Scale bars = 50 µm. E) Representative images of TUNEL assay of testis sections of Control and *Mettl16* ^iKO^ mice at P10, P14, and P18 are shown. Scale bars = 50 µm. The images in the right‐upper corner were the magnified images of the region in white dashed box. F) Quantification of TUNEL‐positive cells per tubule of juvenile Control (Ctrl) and *Mettl16* ^iKO^ (iKO) mice at P10, P14, and P18 are shown. The indicated number of tubules counted from three Ctrl and iKO mice are shown at the bottom of the plots, respectively. ****P < 0.0001. G,H) Immunofluorescence staining (G) and quantification (H) of SYCP3 in testis sections from Control and *Mettl16* ^iKO^ mice at P20 are shown. The quantified data were presented as mean ± SEM. ****P < 0.0001. Scale bars = 50 µm. I) Representative images of nuclear spread analysis of γH2AX in spermatocytes from Control and *Mettl16* ^iKO^ mice at P10, P14, and P18 are shown. P10 testes were used for leptotene/early zygotene detection, P14 testes were used for late zygotene/early pachytene detection, while P18 testes were used for mid‐late pachytene/diplotene detection. Scale bars = 10 µm. White arrowheads indicate autosomal γH2AX signal in pachytene and diplotene spermatocytes. J) Quantification of different type of spermatocytes in Control and *Mettl16* ^iKO^ mice at P18. The quantified data were presented as mean ± SEM. 300 spermatocytes were counted from four Ctrl and iKO mice, respectively. *P < 0.05, ***P < 0.001. K) The quantification of γH2AX signal in leptotene to pachytene spermatocytes of Control and *Mettl16* ^iKO^ mice. Abbreviation: A.U., Arbitrary Units.

Considering that adult tamoxifen‐induced *Mettl16^flox/^* ^−^ *Ddx4*‐*Cre* ^ERT2^ mice could only be used to investigate the role of METTL16 in meiocytes after the first wave of spermatogenesis, we used juvenile *Mettl16^flox/−^Ddx4*‐*Cre* ^ERT2^ mice to elucidate the role of METTL16 during the first wave of spermatogenesis. Intraperitoneal tamoxifen injection into *Mettl16^flox/−^Ddx4*‐*Cre* ^ERT2^ mice at P7 was performed for three consecutive days, and the testes of P10, P12, P14, P16, P18, and P20 mice were harvested for investigation (**Figure** **5**A). Using chromosome spread staining with SYCP3 and METTL16, we found that knockout efficiency of METTL16 in spermatocytes was ≈40% at P10, and the efficiency peaked at P14 (≈80%) and declined at P18 (≈20%) (Figure 5B). The ratios of testis/body weight (mg/g) were significantly lower in *Mettl16* ^iKO^ mice than in control mice from P12 onward (Figure 5C). Consistent with this result, histological analysis showed impaired meiotic progression and numerous apoptotic spermatocytes in the seminiferous tubules, whereas at P20, few spermatocytes and no haploid germ cells were found in *Mettl16* ^iKO^ mice, indicating meiotic arrest (Figure 5D). The apoptotic defects observed in P10, P14, and P18 *Mettl16* ^iKO^ mice were verified and quantified using TUNEL staining (Figure 5E,F). In P20 *Mettl16* ^iKO^ mice, the number of SYCP3‐positive spermatocytes was significantly lower than that in control mice (Figure 5G,H). These data imply that METTL16 is also required for meiotic progression during the first wave of spermatogenesis.

To further explore the function of METTL16 in meiosis in juvenile male mice, we performed chromosome spread assays using P10, P14, P16, and P18 testes from control and *Mettl16* ^iKO^ mice based on the SYCP3 signal as the stage assignment.^[^ ^41^ ^]^ Notably, POL II signals were found to be enriched on the sex chromosomes of P18 pachytene spermatocytes, indicating that the MSCI was also impaired in juvenile *Mettl16* ^iKO^ mice (Figure S6C, Supporting Information). Compared to control mice at P18, the proportions of leptotene (≈13.7% in iKO vs. ≈9.0% in Ctrl), zygotene (≈35.7% in iKO vs. ≈27.0% in Ctrl), and early pachytene cells (≈24.3% in iKO vs. ≈18.0% in Ctrl) were increased in *Mettl16* ^iKO^ mice at P18, while the proportions of mid‐late pachytene (≈14.0% in iKO vs. ≈22.0% in Ctrl) and diplotene (≈12.3% in iKO vs. ≈24.0% in Ctrl) were reduced (Figure 5I,J), indicating that meiotic progression was disrupted in juvenile *Mettl16* ^iKO^ male mice. Interestingly, we observed an increased presence of fragmented dots or aggregated signals of γH2AX on leptotene spermatocytes in *Mettl16* ^iKO^ mice, whereas it was normal in adult *Mettl16* ^iKO^ mice (Figure 5I). This reduced signal of γH2AX was gradually restored and showed no difference between control and *Mettl16* ^iKO^ mice at late zygotene, indicating reduced DSB formation during early meiosis in *Mettl16* ^iKO^ mice (Figure 5I,K). In addition, similar to adult T4 *Mettl16* ^iKO^ mice (Figure 1H), juvenile *Mettl16* ^iKO^ mice showed an abnormal accumulation of the γH2AX signal on the autosomes of pachytene and diplotene spermatocytes (Figure 5I). To further elucidate how METTL16 affects DSB formation, we analyzed the IP‐MS data. IP‐MS and Co‐IP experiments revealed that METTL16 may interact with FUS and EWSR1 in spermatocytes (Figure S6D and Table S1, Supporting Information). Since FUS and EWSR1 are involved in PRDM9 guidance and SPO11/TOPOVIBL cleavage activity,^[^ ^42^, ^43^, ^44^ ^]^ we speculated that METTL16 may also affect SPO11/TOPOVIBL cleavage activity via this interaction. Interestingly, using P10 testes from control and *Mettl16* ^iKO^ mice to detect the levels of FUS and EWSR1 with SYCP3 (marker of spermatocytes) as normalization, we found decreased levels of FUS, in testis lysate from *Mettl16* ^iKO^ mice compared to controls (Figure S6E, Supporting Information). Furthermore, METTL16 has been reported to interact with the MRN complex in somatic cells,^[^ ^25^ ^]^ and during meiosis, the MRN complex can remove SPO11 oligo and assist in the deposition of repair proteins to single‐strand DNA (ssDNA).^[^ ^45^, ^46^ ^]^ Thus, we assayed the interaction between METTL16 and the MRN complex in P12 testes using METTL16 antibody. The results showed no interaction between METTL16 and the MRN complex in the testes, which differed from the results in somatic cells (Figure S6F, Supporting Information). We also measured the expression profile of the MRN complex by IF assays in spermatocytes of control and *Mettl16* ^iKO^ mice, and interestingly, the dynamic expression of MRN complex in *Mettl16* ^iKO^ mice was consistent with IF analyses of γH2AX; that is, a reduced signal intensity from leptotene to early‐mid zygotene, and a normal one in late zygotene (Figure S6G–J, Supporting Information). These data showed that METTL16 is essential for DSB formation in early meiotic prophase I spermatocytes during the first wave of spermatogenesis.

### 2.6 Loss of METTL16 Leads to Impaired Meiotic Recombination, Crossover Formation, and SYCP1 Deposition in Juvenile Mice

**Figure 6:**
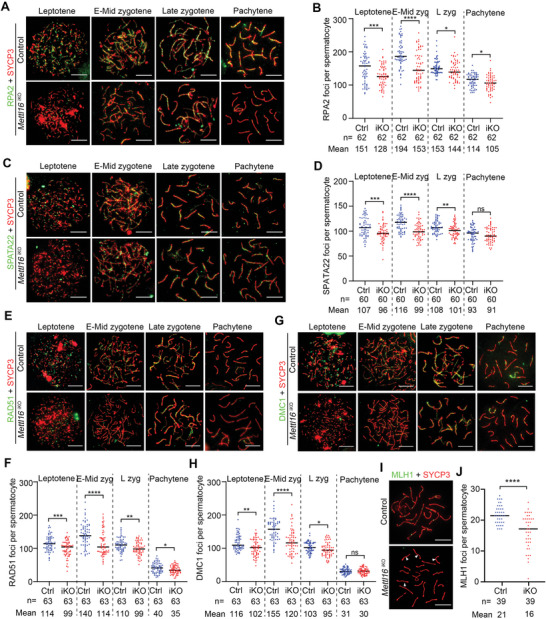
METTL16 is essential for homologous recombination and crossover. A–H). Representative images of nuclear spread analysis A,C,E, and G) and quantification B,D,F, and H) for RPA2 A,B), SPATA22 C,D), RAD51 E,F), and DMC1 G,H) in spermatocytes from Control (Ctrl) and *Mettl16* ^iKO^ (iKO) mice at P10, P14, and P18 are shown. The indicated number of spermatocytes counted from three Ctrl and iKO mice are shown at the bottom of the histogram, respectively. The quantified data were presented as mean ± SEM. Abbreviation: E‐Mid zyg, early to mid‐zygotene; L zyg, late‐zygotene. *P < 0.05, **P < 0.01, ***P < 0.001, ****P < 0.0001. ns, not significant. Scale bars = 10 µm. I,J) Representative images of nuclear spread analysis I) and quantification J) for MLH1 in pachytene spermatocytes from Ctrl and iKO mice at P16‐18 are shown. White arrowheads indicate bivalents without MLH1 foci. The quantified data were presented as mean ± SEM. ***P < 0.001. n = 4 mice. Scale bars = 10 µm.

During meiosis, ssDNA‐binding proteins (RPA2, MEIOB, and SPATA22) and recombinases (DMC1 and RAD51) are the main elements involved in the activation of homologous recombination upon DSB formation to repair genotoxic DSB.^[^ ^47^, ^48^, ^49^, ^50^ ^]^ Thus, we determined the number of RPA2 and SPATA22 foci to probe the nature of the meiotic block in *Mettl16* ^iKO^ juvenile mice using chromosome spread analysis. Compared with control mice, the number of RPA2 foci was significantly reduced in leptotene (≈128 in iKO vs. ≈151 in Ctrl), early‐mid zygotene (≈153 in iKO vs. ≈194 in Ctrl), late zygotene (≈144 in iKO vs. ≈153 in Ctrl), and pachytene spermatocytes (≈105 in iKO vs. ≈114 in Ctrl) of *Mettl16* ^iKO^ juvenile mice (**Figure** **6**A,B), suggesting homologous recombination was impaired upon loss of METTL16 in juvenile mice. Consistently, SPATA22 foci were also reduced in leptotene to late zygotene spermatocytes but did not show a significant difference in pachytene spermatocytes (Figure 6C,D). In addition, consistent with ssDNA‐binding protein signals, the number of RAD51 and DMC1 foci in *Mettl16* ^iKO^ juvenile mice was significantly reduced in leptotene (≈99 in iKO vs. ≈114 in Ctrl and ≈102 in iKO vs. ≈116 in Ctrl, respectively), early‐mid zygotene (≈114 in iKO vs. ≈140 in Ctrl and ≈120 in iKO vs. ≈155 in Ctrl, respectively), and late zygotene spermatocytes (≈99 in iKO vs. ≈110 in Ctrl and ≈95 in iKO vs. ≈103 in Ctrl, respectively), compared with that of control mice (Figure 6E–H). However, only RAD51, but not DMC1, displayed reduced levels in the pachytene spermatocytes of *Mettl16* ^iKO^ juvenile mice (Figure 6E–H). At mid‐pachytene, homologous chromosomes undergo crossover to guide subsequent correct chromosome segregation. MutL homolog 1 (MLH1) marks crossovers, and there are usually 1–2 MLH1 foci in each bivalent.^[^ ^51^ ^]^ In the mid‐pachytene spermatocytes of *Mettl16* ^iKO^ mice, the average number of MLH1 foci was reduced to ≈16, whereas in control mid‐pachytene spermatocytes, ≈21 foci were detected (Figure 6I,J). Taken together, these results suggest that METTL16 ensures correct homologous recombination and crossover during the first wave of spermatogenesis.

To further elucidate the role of METTL16 in meiotic progression during the first wave of spermatogenesis, we examined chromosomal synapsis by nuclear spread analysis of SYCP1, a transverse filament (TF) component of the synaptonemal complex (SC), in leptotene, zygotene, pachytene, and diplotene spermatocytes from juvenile control and *Mettl16* ^iKO^ mice (Figure S7A–E, Supporting Information). Absent or dot‐like SYCP1 signals occupied a significantly higher proportion of control leptotene spermatocytes than did *Mettl16* ^iKO^ leptotene spermatocytes (≈89.7% in Ctrl vs. ≈66.1% in iKO). Correspondingly, the percentage of leptotene spermatocytes with short stretches of SYCP1 was significantly increased in *Mettl16* ^iKO^ leptotene spermatocytes compared to controls (≈33.9% in iKO vs. ≈10.3% in Ctrl) (Figure S7A,E, Supporting Information). Interestingly, SYCP1 was also found to localize to the unsynapsed homologs in *Mettl16* ^iKO^ zygotene and diplotene spermatocytes. The percentage of this cell type was 32.1% in *Mettl16* ^iKO^ zygotene spermatocytes and 1.0% in controls, whereas it was 26.2% in *Mettl16* ^iKO^ diplotene spermatocytes and 11.6% in controls (Figure S7B,D,E, Supporting Information). Additionally, a discontinuous SYCP1 signal was observed on the homologous chromosomes of *Mettl16* ^iKO^ zygotene and diplotene spermatocytes. The percentage of this cell type was 15.4% in *Mettl16* ^iKO^ zygotene spermatocytes and 8.2% in controls, whereas it was 14.1% in *Mettl16* ^iKO^ diplotene spermatocytes and 5.0% in controls (Figure S7B,D,E, Supporting Information). Moreover, the percentage of cells with both types of abnormalities was 3.0% in *Mettl16* ^iKO^ zygotene spermatocytes and 0% in controls, whereas it was 4.6% in *Mettl16* ^iKO^ diplotene spermatocytes and 1.3% in controls (Figure S7B,D,E, Supporting Information). In pachytene spermatocytes, X and Y chromosomes undergo partial synapses in the pseudoautosomal region (PAR). However, colocalization of SYCP3 and SYCP1 beyond the PAR of the sex chromosomes was also detected in juvenile *Mettl16* ^iKO^ mice (Figure S7C, Supporting Information). The percentage in *Mettl16* ^iKO^ pachytene spermatocytes was ≈59.7%, whereas it was only 11.3% in the control mice (Figure S7C,E, Supporting Information). To determine whether there was a complete SC localization for unsynapsed homologs, we examined the localization of SYCE1 and SYCE3, both of which are components of SC central elements (CEs), by microscopy. SYCE1 and SYCE3 signals showed no differences between the spermatocytes of juvenile Ctrl and *Mettl16* ^iKO^ mice (Figure S7F,G, Supporting Information). These results suggest that in *Mettl16* ^iKO^ spermatocytes, abnormal localization is limited to SYCP1, and not the entire SC.

### 2.7 METTL16 Regulates m^6^A Level of Meiosis‐Related Gene *Ubr2*

**Figure 7:**
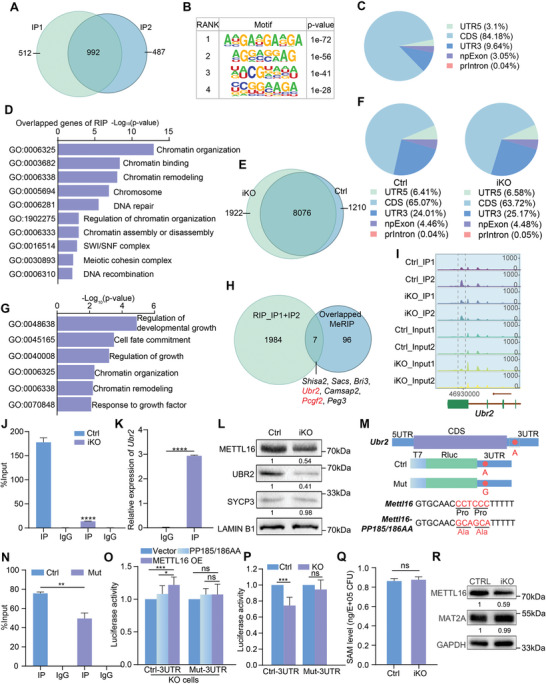
METTL16‐directed m^6^A modification mediates the expression of the meiosis‐related gene *Ubr2*. A) Venn diagram showing the overlap of METTL16 targeted genes between two biological replicates identified by METTL16 RIP‐seq (RIP‐sequencing) in isolated spermatocytes. B) The top four enriched motifs of RIP‐seq data are shown along with *P* values analyzed by HOMER. C) Pie chart of the distribution of METTL16 binding sites in the RNAs based on RIP‐Seq data. D) Gene ontology (GO) analysis of overlapping targeted genes identified by RIP‐Seq data. E) Comparison of genes with m^6^A peaks between Ctrl and iKO spermatocytes determined by MeRIP‐Seq data. F) Pie chart of the distribution of m^6^A peaks in Ctrl and iKO spermatocytes. G) Gene ontology (GO) analysis of the genes with decreased m^6^A peaks. H) Venn diagram showing the overlap of METTL16 targeted genes and genes with decreased m^6^A peaks in both two biological replicates of MeRIP‐seq. The red genes are highly associated with male meiosis in mice. I) IGV plot of m^6^A peaks in *Ubr2* transcript determined by MeRIP‐Seq. J) MeRIP‐qPCR for validation of decreased m^6^A peaks in *Ubr2* in iKO spermatocytes. The site for qPCR analysis was marked by grey dashed line in (I). K) RIP‐qPCR for validation of *Ubr2* as a target of METTL16. L) The protein level of UBR2 in Ctrl and iKO spermatocytes. M) Schematics of m^6^A mutant luciferase vector and mutant overexpression vector construction. N) MeRIP‐qPCR for validation of decreased m^6^A peaks on *Ubr2* transcript in Mutant (Mut) group. **P < 0.01. O) Luciferase activities of control (Ctrl) and Mut 3′UTR vectors with exogeneous overexpression of CMV‐3x Flag (Vector), 3x Flag‐METTL16‐PP185/186AA, and 3x Flag‐METTL16 in *Mettl16* KO GC2 cells. Abbreviation: OE, overexpression. ***P < 0.001, *P < 0.05. ns, not significant. P) Luciferase activities of Ctrl or Mut 3′UTR vectors in Ctrl and KO GC2 cells. ***P < 0.001. ns, not significant. Q) SAM level (Total amount/cell number) within isolated spermatocytes of Ctrl and iKO mice. ns, not significant. R) The protein level of MAT2A in Ctrl and iKO spermatocytes.

To explore the molecular mechanisms underlying the regulation of meiosis by METTL16, we first performed RNA Immunoprecipitation sequencing (RIP‐seq) analysis using METTL16 antibody in isolated spermatocytes. Two IP replicates revealed 992 overlapping genes (**Figure** **7**A; Figure S8A,B and Table S3, Supporting Information), and the motif “UGAAGA” was found to be bound by METTL16 with top rank consistent with that in somatic cells^[^ ^28^ ^]^ (Figure 7B), indicating a high reliability of the sequencing data. The distribution of the binding analysis showed that the METTL16‐binding regions of genes were mainly located on CDS, and further GO analysis of METTL16‐target genes displayed a high relationship with chromatin organization, DNA repair, chromatin remodeling, recombination, etc. (Figure 7C,D; Figure S8C, Supporting Information). Since METTL16 has been reported to be an m^6^A writer,^[^ ^52^ ^]^ we investigated the m^6^A profiling of RNA in spermatocytes by Methylated RNA Immunoprecipitation sequencing (MeRIP‐seq) analysis of spermatocytes isolated from control and *Mettl16* ^iKO^ P14 mice. A total of 18744 and 19983 m^6^A peaks were identified from control and *Mettl16^iKO^* spermatocytes, respectively, using two replicates (Figure S8D,E and Table S4, Supporting Information). Interestingly, we found a total of 9286 and 9998 genes with m^6^A peaks in control and *Mettl16* ^iKO^ spermatocytes, respectively, of which 1922 new genes with m^6^A peaks were identified in *Mettl16* ^iKO^ spermatocytes (Figure 7E and Table S4, Supporting Information). The m^6^A peaks identified in both control and *Mettl16* ^iKO^ spermatocytes were mainly enriched in the CDS and 3′UTR of genes (Figure 7F and Table S5, Supporting Information), and the overall m^6^A profiling did not show obvious differences between *Mettl16* ^iKO^ spermatocytes and controls (Figure S8F, Supporting Information). Further motif analysis revealed that in both control and *Mettl16* ^iKO^ spermatocytes, the classical m^6^A motif RRACH ranked near the top (Figure S8G, Supporting Information) and the motif UACAGAGAA, which was the preferred target of METTL16, also ranked near the top among the motifs of differentially expressed peaks (Figure S8G, Supporting Information), suggesting that METTL16 depletion may affect the m^6^A modification of some transcripts in spermatocytes. GO analysis of genes with downregulated peaks in iKO spermatocytes were associated with regulation of developmental growth, cell fate, chromatin organization, chromatin remodelling, etc. (Figure 7G).

Next, we compared RIP‐seq data with MeRIP‐seq data and found that 7 of the overlapping genes between two replicates with significantly reduced m^6^A peaks were targeted by METTL16 (Figure 7H). Among these 7 genes, *Ubr2* ^[^ ^53^, ^54^, ^55^, ^56^ ^]^ and *Pcgf2* ^[^ ^57^ ^]^ were reported to be involved in male meiosis. Comparing the RIP‐seq data with all genes harboring significantly decreased m^6^A peaks in the MeRIP‐seq data, 56 genes were identified as being targeted by METTL16 (Figure S8H, Supporting Information). Among them, *Ctcf*,^[^ ^58^ ^]^ *Syne2*,^[^ ^59^ ^]^ *Ubr2*,^[^ ^53^, ^54^, ^55^, ^56^ ^]^ and *Anks1* ^[^ ^60^ ^]^ were reported to be involved in male meiosis (Figure S8H, Supporting Information). Based on these analyses, we chose *Ubr2* for further validation, since it is critical for chromatin integrity, SPO11‐dependent DSB formation, and homologous recombination in male meiosis. MeRIP‐qPCR validated the reduced m^6^A level in *Ubr2* transcript and RIP‐qPCR validated that *Ubr2* is the target of METTL16 (Figure 7I–K). In addition, we found that the protein level of UBR2 was also significantly reduced in iKO spermatocytes (Figure 7L). To confirm these results, we generated METTL16‐knockout (KO) GC2 cells (mouse spermatocyte cell line) using the CRISPR/Cas9 strategy and found that both METTL16 and UBR2 protein levels were significantly reduced in the *Mettl16* KO cells (Figure S8I, Supporting Information). Further RIP‐qPCR also revealed that *Ubr2* was a target of METTL16 in GC2 cells (Figure S8J, Supporting Information). To investigate whether the reduction of UBR2 protein was indeed due to reduced m^6^A levels directed by METTL16, we designed and constructed a mutant 3‘UTR luciferase reporter by replacing the specific adenosine (A) in the m^6^A motif with guanine (G) based on the control (Ctrl) 3′UTR luciferase reporter in the *Ubr2* gene, and a METTL16‐PP185/186AA (inactivated m^6^A transferase activity site) mutant overexpression vector (Figure 7M). We then performed MeRIP‐qPCR and found a reduced level of m^6^A on the *Ubr2* transcript in the mutant 3′UTR group (Figure 7N). Dual luciferase assays in both Ctrl and KO GC2 cells showed that METTL16 could not promote the luciferase activity of the reporter construct carrying the mutated 3′UTR and that the luciferase increase in the Ctrl 3′UTR was more pronounced in METTL16 overexpressing cells than in the METTL16‐PP185/186AA group (Figure 7O; Figure S8K, Supporting Information). For further validation, we transfected Ctrl 3′UTR and mutant 3′UTR luciferase reporters into Ctrl and KO GC2 cells, respectively. The results showed that knockout of METTL16 in GC2 cells could significantly reduce luciferase activity only in the Ctrl 3′UTR of the *Ubr2* gene, but not in the mutant one (Figure 7P). Western blot analysis also confirmed a higher level of UBR2 in METTL16‐overexpressing cells than in the METTL16‐PP185/186AA group (Figure S8I, Supporting Information). Together, these data demonstrate that METTL16 targets the *Ubr2* transcript and regulates its m^6^A modification to mediate *Ubr2* expression.

Given that METTL16 has the ability to modulate intracellular SAM homeostasis via the SAM synthetase MAT2A in HEK293T cells,^[^ ^23^ ^]^ we performed SAM measurement assays using LC‐MS analysis of isolated spermatocytes from control and *Mettl16* ^iKO^ mice to investigate whether METTL16 affects SAM levels in spermatocytes. The results showed that SAM levels did not show a significant difference between control and *Mettl16* ^iKO^ spermatocytes (Figure 7Q). Consistently, we found that the protein level of MAT2A in spermatocytes or GC2 cells was not altered by METTL16 loss or overexpression, although METTL16 could target the *Mat2a* transcript in these cells (Figure 7R; Figure S8I,L, Supporting Information), suggesting a distinct function of METTL16 in SAM regulation in mouse spermatocytes and human somatic cells. We also investigated the relationship between METTL16 and METTL3/14 (two classical m6A methyltransferases involved in spermatogenesis). Interestingly, no interaction was observed between METTL16 and METTL3/14, and METTL3/14 protein expression was not affected in *Mettl16* ^iKO^ spermatocytes or GC2 KO cells (Figure S8M–O, Supporting Information). Furthermore, the RIP‐seq data also showed that METTL3/14 was not a target of METTL16 (Table S3, Supporting Information), possibly indicating no redundant function between these three m^6^A methyltransferases. Altogether, these results suggest that METTL16 plays a critical role in m^6^A modification of meiosis‐related genes, but may not have a function in SAM homeostasis in mouse spermatocytes.

### 2.8 METTL16 Interacts with Translation Factors to Regulate the Translation Efficiency of Essential Meiotic Genes

**Figure 8:**
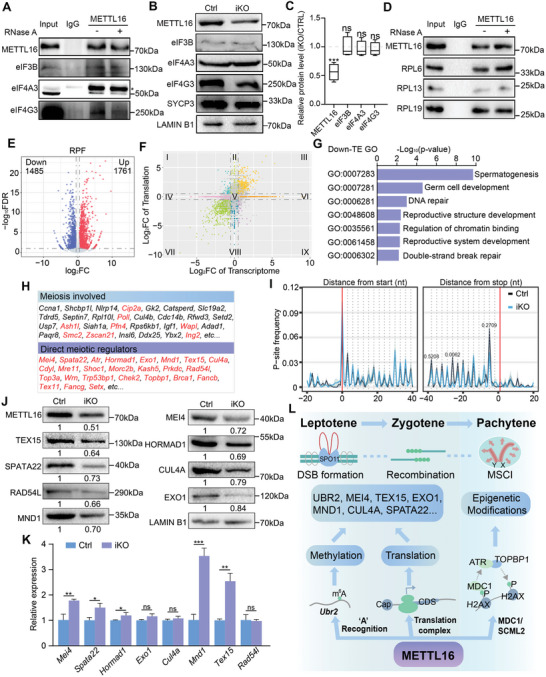
METTL16 interacts with translational factors to regulate translation efficiency of meiosis‐related genes. A) Immunoprecipitation (Co‐IP) assay to validate the interaction between METTL16, eIF3B, eIF4A3 and eIF4G3 using METTL16 antibody with or without RNase A. B) The protein levels of eIF3B, eIF4A3, and eIF4G3 in Ctrl and iKO spermatocytes. n = 15 mice. LAMIN B1 serves as loading control. The asterisk labelled non‐specific band. C) The quantification of METTL16, eIF3B, eIF4A3 and eIF4G3 in isolated spermatocytes from Control (Ctrl) and *Mettl16* ^iKO^ (iKO) juvenile mice. n = 15 mice. ns, not significant, ***P < 0.001. D) Immunoprecipitation (Co‐IP) assay to validate the interaction between METTL16, RPL6, RPL13, and RPL19 using METTL16 antibody with or without RNase A. E) Volcano plot showing differential ribosome‐protected mRNA fragments (RPFs) identified by Ribo‐seq analysis. F) Scatterplot showing fold changes in FPKM at the RNA and RPF levels due to METTL16 deletion. Mode I, up−regulated at Translation level and down−regulated at Transcriptome level; Mode II, up−regulated at Translation level and unchanged at Transcriptome level; Mode III, up−regulated at Translation and Transcriptome level; Mode IV, unchanged at Translation level and down−regulated at Transcriptome level; Mode V, unchanged at Translation and Transcriptome level; Mode VI, unchanged at Translation level and up−regulated at Transcriptome level; Mode VII, down−regulated at Translation and Transcriptome level; Mode VIII, down−regulated at Translation level and unchanged at Transcriptome level; Mode IX, down−regulated at Translation level and up−regulated at Transcriptome level. G) Gene ontology (GO) analysis of the genes with decreased translation efficiency (TE). H) Genes involved in meiosis (black and red) or DSB formation/recombination (red) directly or indirectly with decreased TE. I) Overlay meta‐profiles of meiosis‐related genes in Ctrl and iKO spermatocytes. J) The protein levels of chosen genes in Ctrl and iKO spermatocytes. n = 15 mice. LAMIN B1 serves as loading control. K) The mRNA levels of chosen genes in Ctrl and iKO spermatocytes. n = 15 mice. *P < 0.05, **P < 0.01, ***P < 0.001. ns, not significant. L) Schematic model showing functions of METTL16 in different substage of male meiotic prophase I.

Given that MeRIP‐seq did not reveal a significant decrease in m^6^A levels of DSB formation/recombination‐related genes, other mechanisms for these severe meiotic defects caused by METTL16 deletion deserve further investigation. To this end, we re‐analyzed the IP‐MS data generated from isolated spermatocytes. A total of 855 METTL16‐interacting candidate proteins were identified and more than 80 candidate interactors of METTL16 were ribosomal proteins (Table S1, Supporting Information). GO analysis of the interacting candidates from IP‐MS showed that some highly enriched pathways were related to translation and ribosome biogenesis (Figure S9A, Supporting Information). A number of translation‐related proteins were identified, including translation initiation proteins (eIF4A3, eIF4G3, and eIF3B), translation elongation proteins (eEF1A1), and translation‐facilitating proteins (FXR1, PABPC1, RTRAF, and PRRC2A), etc. (Table S1, Supporting Information). These bioinformatic analyses have raised the possibility of a role for METTL16 in translational regulation in male meiosis. The RNA‐independent interactions of METTL16 with the translation factors eIF3B/eIF4A3/eIF4G3 or the ribosomal proteins RPL6/RPL13/RPL19 were verified in isolated spermatocytes (**Figure** **8**A–D), whereas no obvious change in the protein abundance of eIF3B/eIF4A3/eIF4G3 was observed in METTL16‐deficient spermatocytes (Figure 8C). In addition, the interactions between METTL16 and these translation‐related proteins were also validated in GC2 cells (Figure S9B, Supporting Information), and the protein levels of these proteins were also unchanged between Ctrl and *Mettl16* KO GC2 cells (Figure S9C, Supporting Information). Furthermore, the HPG click assay used to detect nascent protein synthesis revealed a significantly reduced level of translation in *Mettl16* KO GC2 cells (Figure S9D,E, Supporting Information), further highlighting the important role of METTL16 in regulating translation efficiency.

To further understand the role of METTL16 in spermatocyte mRNA translation, we performed ribosome profiling sequencing (Ribo‐seq) on isolated spermatocytes from P15 control (Ctrl) and *Mettl16* ^iKO^ (iKO) mice. Over 10 000 genes were identified by Ribo‐seq (TPM > 1) in Ctrl and iKO spermatocytes (Figure S9F, Supporting Information), and the comparison of Ctrl or iKO replicates showed a high Pearson correlation (Figure S9G, Supporting Information), indicating a high reliability of the sequencing data. In both Ctrl and iKO samples, the 3‐nt periodicity was observed at the peak size of ≈28–30 nt (Figure S9H, Supporting Information), and the mapped reads showed high coverage in the CDS region (Figure S9I, Supporting Information). Ribosome profiling analyses identified 1761 upregulated and 1485 downregulated ribosome‐protected mRNA fragments (RPFs) in METTL16‐depleted spermatocytes (Figure 8E and Table S6, Supporting Information), while the RPFs in the iKO group were greatly reduced at codons near the stop site (Figure S9J, Supporting Information). Codon usage frequency analysis revealed that AAA (lysine) usage at the A site was decreased in the iKO group (Figure S9K, Supporting Information). In addition, we overlapped the translatome and transcriptome of Ctrl and *Mettl16* ^iKO^ spermatocytes and found 1812 genes with downregulated translation efficiency (TE) (Figure 8F and Table S7, Supporting Information). GO term analysis revealed that the genes with downregulated translation efficiency were involved in spermatogenesis, germ cell development, DNA repair, reproductive structure development, regulation of chromatin binding, reproductive system development, and double‐strand break repair (Figure 8G).

Next, among the genes with downregulated TE, we focused on those involved in meiosis. Several genes associated with meiosis were identified, such as *Ccna1*, *Shcbp1l*, *Nlrp14*, *Cip2a*, *Gk2*, *Catsperd*, *Slc19a2*, *Tdrd5*, *Septin7*, *Rpl10l*, *Poll*, *Cul4b*, *Cdc14b*, *Rfwd3*, *Setd2*, *Usp7*, *Ash1l*, *Siah1a*, *Pfn4*, *Rps6kb1*, *Igf1*, *Wapl*, *Adad1*, *Paqr8*, *Smc2*, *Zscan21*, *Insl6*, *Ddx25*, *Ybx2*, and *Ing2*, etc. Among them, *Cip2a, Poll, Ash1l, Wapl*, and *Zscan21*, etc. were closely related to DSB formation/recombination, whereas *Ing2, Smc2*, and *Pfn4*, etc. were related to chromatin organization/accessibility. Most importantly, several classical meiotic regulators were identified that are directly involved in meiotic DSB formation and resection, DNA damage sensing and homologous recombination, such as *Mei4*, *Spata22*, *Atr*, *Hormad1*, *Exo1*, *Mnd1*, *Tex15*, *Cul4a*, *Cdyl*, *Mre11*, *Shoc1*, *Morc2b*, *Kash5*, *Prkdc*, *Rad54l*, *Top3a*, *Wrn*, *Trp53bp1*, *Chek2*, *Topbp1*, *Setx*, *Brca1*, *Fancb*, *Tex11*, and *Fancg*, etc (Figure 8H). The RPFs of these meiosis‐related genes in the iKO group were dysregulated, specifically slightly enriched at the codons near the start site and apparently decreased near the stop site (Figure 8I), suggesting that METTL16 deletion led to abnormal polyribosome occupancy in these genes and ultimately downregulated translation. A number of critical meiotic regulators were selected for validation, including *Mei4, Spata22, Hormad1, Exo1, Mnd1, Tex15, Cul4a*, and *Rad54l*. Their protein levels were all reduced in *Mettl16* ^iKO^ spermatocytes (Figure 8J), while their mRNA levels were increased or unchanged (Figure 8K). These results highlight a critical role for METTL16 as a TE regulator to mediate the translation of essential meiotic proteins in spermatocytes.

## 3 Discussion

Global mutation of *Mettl16* in mice causes embryonic developmental arrest around the time of implantation,^[^ ^30^ ^]^ and conditional knockout of *Mettl16* at E15.5 using *Ddx4*‐*Cre* results in severe germ cell loss and a Sertoli cell‐only phenotype.^[^ ^24^ ^]^ Consistent with the previous study of *Ddx4‐Cre*‐cKO, we also conditionally inactivated *Mettl16* using consecutively expressed *Ddx4‐Cre*, and found a Sertoli cell‐only phenotype from P12 onwards. Further analysis of P3, P5, and P7 *Ddx4‐Cre*‐cKO testes revealed the essential role of METTL16 in spermatogonial maintenance and differentiation. Given that spermatogenesis is arrested at the pre‐meiotic stage using global knockout and the classical *Cre* strategy, in the current study, we used an inducible inactivation approach to uncover the functions of METTL16 in meiosis. Our findings, for the first time, show that the ablation of METTL16 in spermatocytes leads to impaired MSCI in pachytene spermatocytes, and reduced DSB formation/recombination and abnormally increased SYCP1 deposition in early meiotic prophase I spermatocytes, demonstrating the physiological function of METTL16 in meiosis in male mice. Interestingly, in spermatocytes of adult *Mettl16* ^iKO^ mice, only defective MSCI was observed, whereas, in juvenile *Mettl16* ^iKO^ mice, the phenotypes were more severe and diverse, which may be due to the specific characteristics of meiocytes in the first wave of spermatogenesis.

Notably, previous genetic studies have demonstrated that the MSCI checkpoint operates at stage IV,^[^ ^32^, ^61^ ^]^ that is, MSCI‐defective spermatocytes were eliminated at mid‐pachytene stage. The current study revealed that at stages IV and beyond (mid‐late pachytene spermatocytes) in adult *Mettl16* ^iKO^ mice (T4, T6, T8, and T10), the extent of pachytene reduction was significantly severe than that at stage I‐III (early pachytene spermatocytes), consistent with MSCI defective phenotype. In addition, in *Mettl16* ^iKO^ mice at T6 and later time points, at stage IX‐XII, the number of leptotene or zygotene spermatocytes showed a slighter decrease compared with pachytene loss, further highlighting the essential role of METTL16 in pachytene spermatocytes. The possibility of pachytene spermatocyte loss observed in *Mettl16* ^iKO^ mice at stages I‐III could be a defect in meiotic initiation, while the severe reduction in pachytene/diplotene spermatocytes at stages IV‐XII was the collective effect of defects in both meiotic initiation and MSCI. Moreover, our RNA‐seq data revealed higher transcription levels on the X and Y chromosomes, while levels in the autosomes were less altered, further indicating the specific role of METTL16 in regulating transcription of genes on the sex chromosomes. METTL16 participates in erythropoiesis by safeguarding genome integrity, specifically by regulating DNA repair‐related *Fancm* and *Brca2* mRNA.^[^ ^62^ ^]^ FANCM and BRCA2 (FANCD1)^[^ ^38^ ^]^ are components of the FA proteins, which are localized at XY body during meiosis and mediate the deposition of H3K9me2 in mid‐late pachytene spermatocytes.^[^ ^39^ ^]^ Interestingly, we did not observe abnormal deposition of H3K9me2 in *Mettl16* ^iKO^ spermatocytes; however, we indeed observed abnormal localization of H2AK119ub, MacroH2A1, USP7, H3K9me1, H3K9AC, and SUMO‐1 in the XY body of mid‐late pachytene spermatocytes in *Mettl16* ^iKO^ mice. The interaction between METTL16 and MDC1/SCML2, which is involved in spreading DDR factors to chromosome‐wide domains and the epigenetic programming of the XY body respectively for MSCI establishment and maintenance,^[^ ^15^, ^17^, ^18^, ^34^ ^]^ further suggests that METTL16 participates in the epigenetic programming in germ cell meiosis in a manner distinct from that in somatic cells.

Furthermore, this study revealed a diverse and interesting phenotype of reduced DSB formation in juvenile *Mettl16* ^iKO^ mice during the first wave of spermatogenesis, which is reminiscent of mutants of DSB formation‐related genes such as *Topo6bl*,^[^ ^63^ ^]^ *Ankrd31*,^[^ ^4^, ^64^ ^]^ and *Sycp2*.^[^ ^8^ ^]^ This reduced DSB formation seems to be gradually compensated for and completed in late zygotene spermatocytes based on γH2AX signal. Intriguingly, we found that METTL16 interacted with FUS and EWSR1, which have previously been reported to regulate PRDM9‐directed SPO11‐mediated DNA breakage.^[^ ^42^, ^43^, ^44^ ^]^ EWSR1 is implicated in guiding PRDM9, the histone methyltransferase, to define H3K4me3‐marked hotspots.^[^ ^42^, ^43^ ^]^ FUS interacts with PRDM9, SPO11, and REC114 to regulate hotspot direction and SPO11/TOPOVIBL cleavage activity.^[^ ^44^ ^]^ We, therefore, hypothesized that the interaction also affects DSB formation by influencing the direction of recombination hotspots and the cleavage activity of SPO11/TOPOVIBL. However, it may be necessary in the future to introduce mice with a mutated interaction domain between these proteins to confirm this hypothesis. In addition, UBR2 is required for mouse spermatocytes to accumulate sufficient SPO11‐dependent recombination.^[^ ^53^, ^54^, ^55^, ^56^ ^]^ In the current study, MeRIP‐seq data and MeRIP‐qPCR showed that m^6^A peaks in *Ubr2* were significantly reduced in METTL16‐deficient spermatocytes. The in vitro luciferase assay further confirmed that this m^6^A modification on the *Ubr2* transcript is essential for its expression, suggesting a critical role for the METTL16‐directed m^6^A modification in DSB formation and recombination. Consistent with the dynamics of γH2AX, IF staining of the MRN complex displayed a similar trend in METTL16‐deficient spermatocytes. However, no interaction between METTL16 and the MRN complex was detected, although this interaction was verified in somatic cells.^[^ ^25^ ^]^ Thus, we speculated that the reduced expression of the MRN complex in leptotene to mid‐zygotene spermatocytes may be due to reduced DNA breakage in juvenile *Mettl16* ^iKO^ mice. In addition, the number of foci of the ssDNA‐binding proteins RPA2 and SPATA22 and the recombinases RAD51 and DMC1 were all reduced from leptotene to late zygotene in juvenile *Mettl16* ^iKO^ mice; however, the extent of reduction was greatly lower compared with the former stages in late zygotene and pachytene (Figure 6). This continuously decreasing DSB repair‐related protein levels in the spermatocytes of *Mettl16* ^iKO^ juvenile mice may be explained by reduced protein levels of repair proteins and never compensated DSB sites on chromosomes. Moreover, we found highly accumulated SYCP1 deposition on unsynapsed chromosomes in *Mettl16* ^iKO^ spermatocytes, while other components of SC complex showed no obvious defects, possibly because of the role of METTL16 in controlling the tendency of SYCP1 to self‐associate.^[^ ^65^, ^66^ ^]^

Notably, the interaction between METTL16 and the translation initiation complex, which was our primary focus, has been previously reported in somatic cells.^[^ ^28^, ^29^ ^]^ The METTL16‐eIF4E2 interaction impedes the recruitment of eIF4E2 to 5′ cap structure to enhance cap recognition by eIF4E and downregulate the translation of key oncogenes.^[^ ^29^ ^]^ METTL16 also cooperates with the translation initiation proteins eIF3a and eIF3b to regulate translation efficiency and tumorigenesis.^[^ ^28^ ^]^ In the current study, IP‐MS revealed possible interactions between METTL16, more than 80 ribosomal proteins, and several translation‐related proteins such as eIF4A3, eIF4G3, and eIF3B. The interactions between METTL16 and eIF3B/eIF4A3/eIF4G3 and ribosomal proteins RPL6/RPL13/RPL19 were validated using Co‐IP assay in spermatocytes. In addition, among the candidate interactors, FXR1, PABPC1, RTRAF, and PRRC2A were all reported to facilitate the translational process. FXR1 interacts with eIF4G3 and PABPC1 to promote translation and drive spermiogenesis.^[^ ^67^ ^]^ PABPC1 is involved in initiating translation during spermatogenesis.^[^ ^68^ ^]^ RTRAF is a component of the cap‐binding complex RTRAF‐HSPC117‐DDX1‐FAM98B, which functions in cytoplasmic mRNA transport and translation.^[^ ^69^ ^]^ m^6^A reader PRRC2A affects meiosis I progression by modulating mRNA degradation and translation efficiency.^[^ ^70^ ^]^ Our Ribo‐seq analysis confirmed that METTL16 is a critical regulator for translation efficiency of essential meiotic genes in early meiotic cells, such as MEI4, SPATA22, HORMAD1, EXO1, MND1, TEX15, CUL4A, and RAD54L etc.^[^ ^11^ ^]^ MEI4 is a member of pre‐DSB machinery for DSB generation.^[^ ^71^ ^]^ SPATA22 is essential for HJ formation and synapsis.^[^ ^48^ ^]^ HORMAD1 is needed for chromosome organization and pre‐DSB machinery recruitment.^[^ ^8^ ^]^ EXO1 is an exonuclease involving in DSB resection.^[^ ^45^ ^]^ MND1 is a recombination mediator protein assisting in stimulating recombinase activity and strand invasion.^[^ ^72^ ^]^ TEX15 functions as a recombination mediator protein required for RAD51/DMC1 loading at DSB sites.^[^ ^73^ ^]^ CUL4A is required for DSB repair.^[^ ^74^ ^]^ RAD54L can dismantle D‐loops formed by RAD51, and is essential for homologous recombination and DSB repair.^[^ ^75^, ^76^ ^]^

Interestingly, various piRNA‐related proteins were identified as candidate interactors of METTL16 in our IP‐MS data, such as RNF17, ADAD2, and TDRD1, suggesting a possible role for METTL16 in regulating RNA processing and the piRNA pathway. In particular, the hypotheses of phase separation, MSCI, and XY body formation have been documented;^[^ ^12^, ^77^ ^]^ in other contexts, SUMOylation has been implicated in phase separation.^[^ ^78^ ^]^ In this study, abnormal SUMOylation in the XY body of *Mettl16* ^iKO^ pachytene spermatocytes was identified, raising the possibility that METTL16 is involved in the SUMO pathway and phase separation during meiosis. Other than these, the role of the classical m^6^A methyltransferase METTL3/14 in spermatogenesis has been reported previously.^[^ ^40^, ^79^ ^]^ In our study, we found that the protein levels of METTL3/14 were not altered in *Mettl16* ^iKO^ spermatocytes or *Mettl16* KO GC2 cells, and no interaction between them was identified. Our RIP‐seq data also revealed that neither *Mettl3* nor *Mettl14* was the target of METTL16, possibly indicating a weak association between METTL16 and METTL3/14. However, the sequencing data of METTL3/14 in germ cells, especially in spermatocytes, was limited, leaving a gap for us to further investigate the relationship between them during male meiosis. Furthermore, in contrast to the results in HEK293T or 293A‐TOA cells,^[^ ^23^, ^80^ ^]^ we found that the levels of SAM and MAT2A were not obviously altered in METTL16‐depleted spermatocytes or GC2 cells, possibly due to the different characteristics between human and mouse cells, or somatic and germ cells. Therefore, how METTL16 controls the methylation process through other pathways, such as affecting modifications on other RNA species in mouse spermatocytes, deserves further investigation. Importantly, in the current mouse model, METTL16 protein levels were significantly reduced in iKO mice, making it difficult to accurately identify the methyltransferase function of METTL16 during male meiosis. Our previous study demonstrated that the PP185‐186AA of METTL16 was necessary for spermatogenesis,^[^ ^81^ ^]^ and our research illustrated that this methylation site was important for UBR2 expression, suggesting that the methyltransferase activity of METTL16 may also be essential for male meiosis. However, further studies using a point mutant mouse model with the PP185‐186AA mutation, which only affects methylation activity rather than protein levels, are needed to investigate its function.^[^ ^23^, ^30^ ^]^ Notably, the phenotypes in juvenile *Mettl16^iKO^* mice were diverse, and it is difficult to conclude which defect was the most significant and critical contributor to meiocyte fate determination because the precise knockout time of each spermatocyte was difficult to determine when using the tamoxifen‐inducible knockout mouse model. In addition, the efficiency of the deletion varied at different time points and in different mice; therefore, the proportion of abnormalities in *Mettl16* ^iKO^ mice was not as severe as in conditional knockout mice, driven by classical *Ddx4‐Cre* and/or *Stra8‐Cre*. Nonetheless, the existing data were sufficient to conclude that METTL16 is essential for meiotic progression.

## 4 Conclusions

In conclusion, our investigation introduced a unique tamoxifen‐induced knockout mouse model to uncover METTL16 as a novel regulator that promotes MSCI establishment and maintenance, DSB formation, homologous recombination and proper SYCP1 localisation to ensure correct meiotic progression. The combined phenotypic and mechanistic results suggest that METTL16 has a broad function during male meiosis. In leptotene or zygotene spermatocytes, METTL16 regulates the m^6^A level of the *Ubr2* transcript and cooperates with translational factors to regulate protein levels of essential meiotic genes (UBR2, MND1, SPATA22, and EXO1, etc.) to control DSB formation and recombination processes. In pachytene spermatocytes, METTL16 cooperates with MDC1/SCML2 to regulate DDR expansion and epigenetic modifications in the XY body for MSCI establishment and maintenance (Figure 8L). The results of the current study provide new insights into the molecular context of METTL16 regulation in meiotic prophase I, providing a more comprehensive understanding of male meiosis and spermatogenesis.

## 5 Experimental Section

### Ethics Statement

All animal procedures were approved by the Institutional Animal Care and Use Committee (IACUC) of Tongji Medical College, Huazhong University of Science and Technology. All mice were housed under specific pathogen‐free (SPF) conditions, in the Laboratory of Animal Center, Huazhong University of Science and Technology. All experiments with mice were conducted ethically according to the Guide for the Care and Use of Laboratory Animal guidelines.

### Mice

All mice were maintained on the C57BL/6J genetic background. *Mettl16^flox/+^* mice were purchased from Cyagen Biosciences company. Two strategies to generate germ cell‐specific *Mettl16* knockout mice were used. The *Ddx4*‐Cre (018 980) and *Ddx4*‐Cre^ERT2^ (024 760) mouse strains were obtained from The Jackson Laboratory. First, to study the role of *Mettl16* in germ cells, *Mettl16^flox/−^ Ddx4*‐*Cre* mice (termed *Mettl16* ^cKO^) was generated, in which the constitutively active *Cre* is expressed in germ cells at embryonic day 15.5 (E15.5). Second, to study the function of *Mettl16* at the meiotic stage during germ cell development, *Mettl16^flox/−^Ddx4*‐*Cre* ^ERT2^ mice (called *Mettl16* ^iKO^ or iKO) was generated. *Ddx4*‐*Cre* ^ERT2^ is a tamoxifen‐inducible *Cre* that induces *Cre*‐mediated deletion of the floxed exons after tamoxifen injection.^[^ ^82^ ^]^ As described previously,^[^ ^83^, ^84^, ^85^ ^]^ for inducible deletion of *Mettl16* in germ cells, tamoxifen (Sigma, cat#T5648) was suspended with corn oil (Sigma, cat#C8267) and injected intraperitoneally daily into 8‐week‐old *Mettl16^flox/−^Ddx4‐Cre* ^ERT2^ males at a dose of 2 mg/30 g body weight for five consecutive days or into juvenile *Mettl16^flox/−^Ddx4‐Cre* ^ERT2^ males (P7) for three consecutive days. *Mettl16^flox/−^* males treated with tamoxifen accordingly were used as controls. For adult *Mettl16* ^iKO^ male mice, testes were collected for analysis at 2, 4, 6, 8, 10, 12, and 60 days post‐tamoxifen treatment (dpt), respectively. For juvenile *Mettl16* ^iKO^ males, testes were collected for analysis at 0, 2, 4, 6, 8, and 10 dpt, corresponding to P10, P12, P14, P16, P18, and P20, respectively. Genotyping of the mice was performed using PCR amplification of genomic DNA extracted from mouse tails. The primer sequences used were listed in Table S8 (Supporting Information).

### Purification of Spermatogenic Cells and Sertoli Cells

STA‐PUT velocity sedimentation was used for purification of different types of germ cells, including spermatogonia, spermatocytes, and round spermatids. To obtain single testicular cell suspension, mouse testes were treated with collagenase IV (Sigma, C5138) and trypsin (Sigma, 9002‐07‐7). After washing with DMEM/F12 medium, the testicular cell suspension was filtered in a linear BSA gradient. Different fractions were collected based on its cell size, and the purity of different types of cells were measured by IF staining, qPCR and western blot assays. Each purification was performed on testes from more than three males. The spermatocyte samples with a purity of more than 80% were used for subsequent sequencing and/or western blot assays.

### Chromatin Fractionation

The nuclear and cytoplasm protein were extracted using Nuclear and Cytoplasmic Protein Extraction Kit (Beyotime Biotechnology, P0027) according to the manufacturer's instructions. Briefly, different types of cells that had been isolated from testes were respectively harvested and dissociated in 200 µl Buffer A with 15 min ice bath. Then, 10 µl buffer B was added into the suspension then mix it thoroughly by 5 s vigorous vortex and 1 min ice bath. Followed by centrifugation at 16 000 g for 5 min at 4 °C, the supernatant was transferred to a clean cold tube as cytoplasm protein. To obtain nuclear protein, the sediment was further resuspended in 50 µl nuclear protein extraction reagent containing 1 mM PMSF. After being vortexed for 15–30 s and ice bathed for 1–2 min in turn for 30 min totally, the mixture was centrifugated at 16 000 g for 10 min at 4 °C and then transferred the supernatant to a clean cold tube as nuclear protein. The subcellular fractions were analyzed by western blot assay.

### Quantitative Real‐Time PCR (qPCR)

RNA was prepared using TRIzol reagent (Invitrogen, 15596‐025) and was reverse transcribed using 1st Strand cDNA Synthesis SuperMix for qPCR (Yeasen, 11141ES60) according to manufacturer's procedures. qPCR was performed using SYBR green master mix in a Step One Plus machine (Applied Biosystems, 4 309 155) or Automatic PCR Analysis System (TianLong, Gentier 96R). Statistics were analyzed using the 2^−△△Ct^ method normalized with *Lamin A* as controls. All primers used for qPCR were listed in Table S8 (Supporting Information).

### Histology, TUNEL, and Immunofluorescence

For histological analysis, mouse testes and epididymides were fixed in Bouin's solution (Sigma, Lot#SLBJ3855V) at room temperature (RT) overnight, embedded in paraffin, and sectioned in 5 µm. Sections were stained with a Periodic Acid‐Schiff (PAS) kit according to the manufacturer's instructions. For TUNEL and immunofluorescence (IF) assays, testes were fixed in 4% paraformaldehyde, dehydrated in sucrose, and embedded in O.C.T (Sakura Finetek, 4583). 5 µm sections were cut, and antigen retrieval was performed using citrate (pH = 6.0). After three times rinsing with PBS, the slides were blocked in 5% normal donkey serum for 1 h at RT. Then, the slides were incubated with primary antibodies and secondary antibodies and mounted with a mounting medium with DAPI for imaging. Detailed information on the antibodies used in this study was provided in Table S9 (Supporting Information).

### Nuclear Spread Analyses

For surface nuclear spread analysis, spermatocytes were fixed to slides according to the method described previously^[^ ^86^ ^]^ with minor modifications. Briefly, after tunica albuginea removal, testicular tubules were separated from the testis and placed in hypotonic extraction buffer (pH = 8.2) containing 30 mM Tris, 50 mM sucrose, 17 mM trisodium citrate dihydrate, 5 mM EDTA, 0.5 mM DTT, and 1 mM PMSF for 2 h. Subsequently, the tubules were smashed repeatedly in 100 µl of 100 mM sucrose buffer (pH = 8.2) to make a cell suspension. The suspension was then spread on slides covered with fixation buffer (pH = 9.2) containing 1% PFA and 0.15% TritonX‐100. After 2 h of incubation in a humidity box at RT, the slides were air‐dried and washed twice with 0.4% Photo‐Flo 200 (Kodak). The thoroughly air‐dried slides were stored at −80 °C for the IF staining. The antibodies used were shown in Table S9 (Supporting Information).

### Immunoprecipitation and Western Blot Analyses

Isolated spermatocytes were transferred into iced WB/IP Lysis buffer (Beyotime, P0013) and were then clarified by centrifugation at 12 000 g. Immunoprecipitation was performed using Protein A/G Magnetic Beads (MCE, HY‐K0202) according to the manufacturer's instructions, followed by western blot analyses. For western blot, the protein lysates were loaded on an SDS/PAGE gel and then electroblotted onto a PVDF membrane (Bio‐Rad). The membranes were then incubated with primary and secondary antibodies and visualized using the ECL solutions (ClarityTM Western ECL Substrate, Bio‐Rad) and ChemiDoc XRS+ system (Bio‐Rad). The antibodies used were listed in Table S9 (Supporting Information).

### RNA‐Sequencing (RNA‐Seq)

RNA of isolated pachytene spermatocytes was extracted using TRIzol reagent (Invitrogen, 15596‐025) according to the manufacturer's instructions. A total amount of 2 µg of RNA per sample was used for the mRNA library, and base pairs were generated by the BGISeq 500 platform. After the removal of adaptor reads and low‐quality reads of raw data via Fastp software, HISAT2 was used to rearrange the sequencing reads based on the MM.GRCm39.109.

### RNA Immunoprecipitation and Sequencing Analysis

RNA immunoprecipitation (RIP) was performed using spermatocytes that were isolated from ≈100 testes of P15 mice by STA‐PUT method. Briefly, after washing the harvested cells using 1x PBS buffer, cell sediment was lysed in lysis buffer (50 mM Tris‐HCl pH = 7.4, 100 mM NaCl, 0.5% NP‐40, 1:100 protease inhibitors cocktail, RNase inhibitor) followed by 20 min ice bath to promote lysis and centrifugation at 15 000 g for 10 min at 4 °C. Then, 10% supernatant was kept as input and the remaining was used in subsequent immunoprecipitation reactions. For each immunoprecipitation reaction, 5 µg METTL16 antibody and Rabbit IgG as control was incubated with Protein A/G Magnetic Beads (MCE, HY‐K0202) in lysis buffer. After agitating at 4 °C for 4 h, control IgG and METTL16 antibody‐coated beads were incubated with spermatocytes lysis extracts and were agitated gently overnight at 4 °C. The next day, the bead complexes containing antibodies, target proteins, and RNA were washed for 50 min at 4 °C and repeated three times. The RNA of input and RIP samples was extracted using TRIzol reagent (Invitrogen, 15596‐025). KC‐Digital TM Stranded mRNA Library Prep Kit for Illumina was used to construct the stranded RNA sequencing library following the manufacturer's instructions. The Kit eliminates duplication bias in PCR and sequencing by using a unique molecular identifier (UMI) to label the pre‐amplified cDNA molecules. The library products corresponding to 200 bp were enriched, quantified, and sequenced on a Novaseq 6000 sequencer (Illumina) with a PE150 model. Trim‐galore was used to filter the raw sequencing data, discarding low‐quality reads and trimming reads contaminated with adaptor sequences. The de‐duplicated consensus sequences were mapped to the UCSC mouse genome reference (GRCm38.87) using STAR software with default parameters. Peak calling was performed using ExomePeak software, and annotation was performed using bedtools. RT‐qPCR primers were designed based on RIP‐seq data.

### Methylated RNA Immunoprecipitation Sequencing (MeRIP‐seq), MeRIP‐qPCR, and Data Analysis

Total RNAs were extracted from isolated spermatocytes using TRIzol reagent (Invitrogen, 15596‐025) following the manufacturer's procedures. After DNA digestion by DNase I, RNA quality was measured by examining A260/A280 with a Nanodrop spectrophotometer (Thermo Fisher Scientific Inc). Then, the qualified RNAs were finally quantified by Qubit 3.0 with a Qubit RNA Broad Range Assay kit (Life Technologies, Q10210). 50 µg total RNAs were used for polyadenylated RNA enrichment by VAHTS mRNA Capture Beads (VAHTS, cat. NO. N401‐01/02). The mixture of 20 mM ZnCl_2_ and mRNA was incubated at 95 °C for 10 min until the RNA fragments were mainly distributed in 100–200 nt, and then 10% was saved as “Input” and the rest was used for m^6^A‐immunoprecipitation (m^6^A‐IP). Input and m^6^A‐IP samples were prepared by TRIzol reagent (Invitrogen, cat. NO 15596‐026), the products were used for sequencing and subsequent qPCR confirmation. The stranded RNA sequencing library was constructed by KC‐Digital Stranded mRNA Library Prep Kit for Illumina (Catalog NO. DR08502, Wuhan Seqhealth Co., Ltd. China) following the manufacturer's instruction. 200–500 bps library products were enriched, quantified, and sequenced on DNBSEQ‐T7 sequencer (MGI Tech Co., Ltd. China) with PE150 model.

Raw sequencing data was first filtered by Trimmomatic (version 0.36) to trim low‐quality reads and contaminated reads. Clean Reads were further treated with in‐house scripts to eliminate duplication bias introduced in library preparation and sequencing. Reads in the same cluster were compared to each other by pairwise alignment, and then those with over 95% sequencing identity were grouped into a new sub‐cluster. After all sub‐clusters were generated, multiple sequence alignment was performed to get one consensus sequence for each sub‐cluster. The de‐duplicated consensus sequences were used for m^6^A site analysis. They were mapped to the reference genome of *Mus musculus* from GRCm38.87 using STAR software (Version 2.5.3a) with default parameters. The exomePeak (Version 3.8), bedtools (Version 2.25.0), and deepTools (Version 2.4.1) software were used for peak calling, annotation, and distribution analysis. The differential m^6^A peaks were identified by a Python script using a Fisher test. Sequence motifs enriched in m^6^A peaks were identified using Homer (Version 4.10). The sites used for qPCR confirmation were based on MeRIP‐sequencing data.

### KO Cell Line, Cell Transfection, Point Mutation Plasmid Construction and Luciferase Reporter Assay

GC2 cells were cultured in DMEM (Procell, PM150210) containing 10% FBS (AlbuminBovine, 4240GR100) and 1% Penicillin‐Streptomycin Solution (Biosharp, BL505A). Cell lines were cultured in a humidified incubator at 37 °C in an atmosphere containing 5% CO_2_. To construct *Mettl16* KO GC2 cells, sgRNA was designed and inserted into lentiviral CRISPR plasmid (Addgene, pXPR_001). sgRNA sequence was shown in Table S9 (Supporting Information).

Part of 3′UTR regions (selected regions: 47239217–47240481) of *Ubr2* were cloned into pCMV‐Rluc‐MCS‐Neo (MiaoLingBio, P54803) with renilla luciferase. For mutant reporter plasmid, the adenosine (A) in m^6^A motif was replaced by guanine (G) (Site: 47 239 605). The proline (Pro) at 185/186 sites of *Mettl16* CDS were replaced with neutral alanine (Ala). For luciferase assay, GC2 cells were seeded into 24‐well plate followed by co‐transfection of wild‐type or mutated *Ubr2* reporter plasmids, 3x Flag, 3x Flag‐METTL16‐PP185/186AA, or 3x Flag‐METTL16, and pGL3‐Control plasmids (firefly luciferase reporter vector) (MiaoLingBio, P0195). After 42 h, cells were harvested to access the luciferase activity using Dual‐Lumi Luciferase Reporter Gene Assay Kit (Beyotime, RG088S) with normalization to pGL3‐Control. All luciferase activity was measured using a microplate reader (BioTek, 17010919).

### Ribosomal Sequencing

A total of 10^^^7 isolated spermatocytes were prepared for cell lysis. RPFs (ribosome‐protected RNA fragments) was extracted using RNA clean&Concentrator‐5 Kit (ZYMO, R1016), while Epi RiboRNA Depletion Kit (Human/Mouse/Rat) (Epibiotek, R1805) was used for rRNA depletion. Ribosome profiling was performed using Epi Ribosome Profiling Kit (Epibiotek, R1814), and libraries were constructed using QIAseq miRNA Library Kit (QIAGEN, 1 103 679). Clean reads between 25 and 35 bp were mapped to the mouse genome and transcriptome (GRCm38) using hisat2. Ribosome profiling was estimated using RiboWaltz package. Read counts were calculated and normalized as RPKM values using DESeq2 package. Translational efficiencies (TE) were determined as the ratio of (normalized abundance determined by ribosome profiling)/(normalized abundance determined by RNA‐seq).

### Nascent Protein Assay

GC2 cells (both WT and KO cells) were harvested and processed using the BeyoClick HPG Protein Synthesis Kit with Alexa Fluor 594 according to the manufacturer's instructions (Beyotime, P1209S). Briefly, cells were cultured on slides and were gently washed with PBS and then incubated in 1x HPG solution for 2 h. After completion of HPG labelling, fixative solution was added for 15 min at RT and removed for subsequent penetration. The rate of nascent protein synthesis was assessed after Click activation with Click working buffer (a mixture of Click Reaction Buffer, CuSO4, Azide 594, and Click Additive Solution), based on the relative fluorescence intensity of HPG/Hochest.

### SAM Measurement

Isolated spermatocytes extracted from Ctrl or iKO mice were resuspended and then adjusted to pH = 4 with formic acid. After ultrasonication with an ice bath for 30 min, following by centrifugation at 12 000 g at 4 °C for 5 min, the supernatant was collected for filtration with CA‐CN, 0.22 µm. SAM (Yuanye Bio‐Tech, 29908‐03‐0) standard solution was prepared at concentrations of 5.0, 2.0, 1.0, 0.5, 0.2, 0.1, and 0.05 nmol mL^−1^. HPLC (Shimadzu, LC‐20AD) and Mass Spectrometer (AB, QTRAP 5500) were used for amount determination. Parameters: Ionization method: ESI+; Air curtain pressure: 35 psi; Spray voltage: 5500 v; Atomizing gas pressure: 60 psi; Auxiliary gas pressure: 60 psi; Atomization temperature: 500.

### Statistical Analysis

All quantitative data were presented as mean ± SEM. Significance was tested using the two‐tailed unpaired Student's *t*‐test (*P < 0.05, ** P < 0.01, *** P < 0.001, and **** P < 0.0001) with Prism 9.0 (GraphPad Software). Data analysis was carried out using RStudio 2023.06.1+524, or Metascape.^[^ ^87^ ^]^ Image processing was done using Photoshop (Adobe) and ImageJ (NIH).

All RNA sequencing data are deposited in the NCBI SRA (Sequence Read Achieve) database with the accession numbers PRJNA1025133 (RNA‐Seq of pachytene spermatocytes, related to Figure 4), PRJNA1025139 (MeRIP‐Seq of spermatocytes, related to Figure 7; Figure S8, Supporting Information), PRJNA1104224 (RIP‐Seq of spermatocytes, related to Figure 7; Figure S8, Supporting Information), and PRJNA1170270 (Ribo‐Seq of spermatocytes, related to Figure 8; Figure S9, Supporting Information).

## Conflict of Interest

The authors declare no conflict of interest.

## Author Contributions

L.Y., N.J., W.X. contributed equally to this work. L.Y. and S.Y. conceived and designed the study. L.Y., N.J., W.X., S.Y., J.Z., M.X., K.L., Y.Z., X.X., Y.G., H.G., T.L., Y.L., X.W., Y.Z., and F.W. performed most bench work and data analyses. Y.G. performed the bioinformatic analysis. L.Y. wrote the manuscript. S.Y. supervised the project and revised the manuscript. All authors read and approved the manuscript.

## Supporting information



Supporting Information

Supporting Information

## Data Availability

The data that support the findings of this study are available from the corresponding author upon reasonable request.
