# Supplementary material for: METTL16 is Required for Meiotic Sex Chromosome Inactivation and DSB Formation and Recombination during Male Meiosis
Source: Adv Sci (Weinh). 2024 Nov 28;12(3):2406332. doi: 10.1002/advs.202406332 (PMC11744674; doi:10.1002/advs.202406332)
Supplement: Supplementary file 1 — Supporting Information [file ADVS-12-2406332-s001.pdf]

ADVANCED SCIENCE
Open Access

Supporting Information

for *Adv. Sci.*, DOI 10.1002/advs.202406332

METTL16 is Required for Meiotic Sex Chromosome Inactivation and DSB Formation and Recombination during Male Meiosis

Lisha Yin, Nan Jiang, Wenjing Xiong, Shiyu Yang, Jin Zhang, Mengneng Xiong, Kuan Liu, Yuting Zhang, Xinxin Xiong, Yiqian Gui, Huihui Gao, Tao Li, Yi Li, Xiaoli Wang, Youzhi Zhang, Fengli Wang* and Shuiqiao Yuan**

Supplementary information

METTL16 IS REQUIRED FOR MEIOTIC SEX CHROMOSOME INACTIVATION AND DSB FORMATION AND RECOMBINATION DURING MALE MEIOSIS

Lisha Yin, et. al.

Figure S1 (Related to Figure 1)

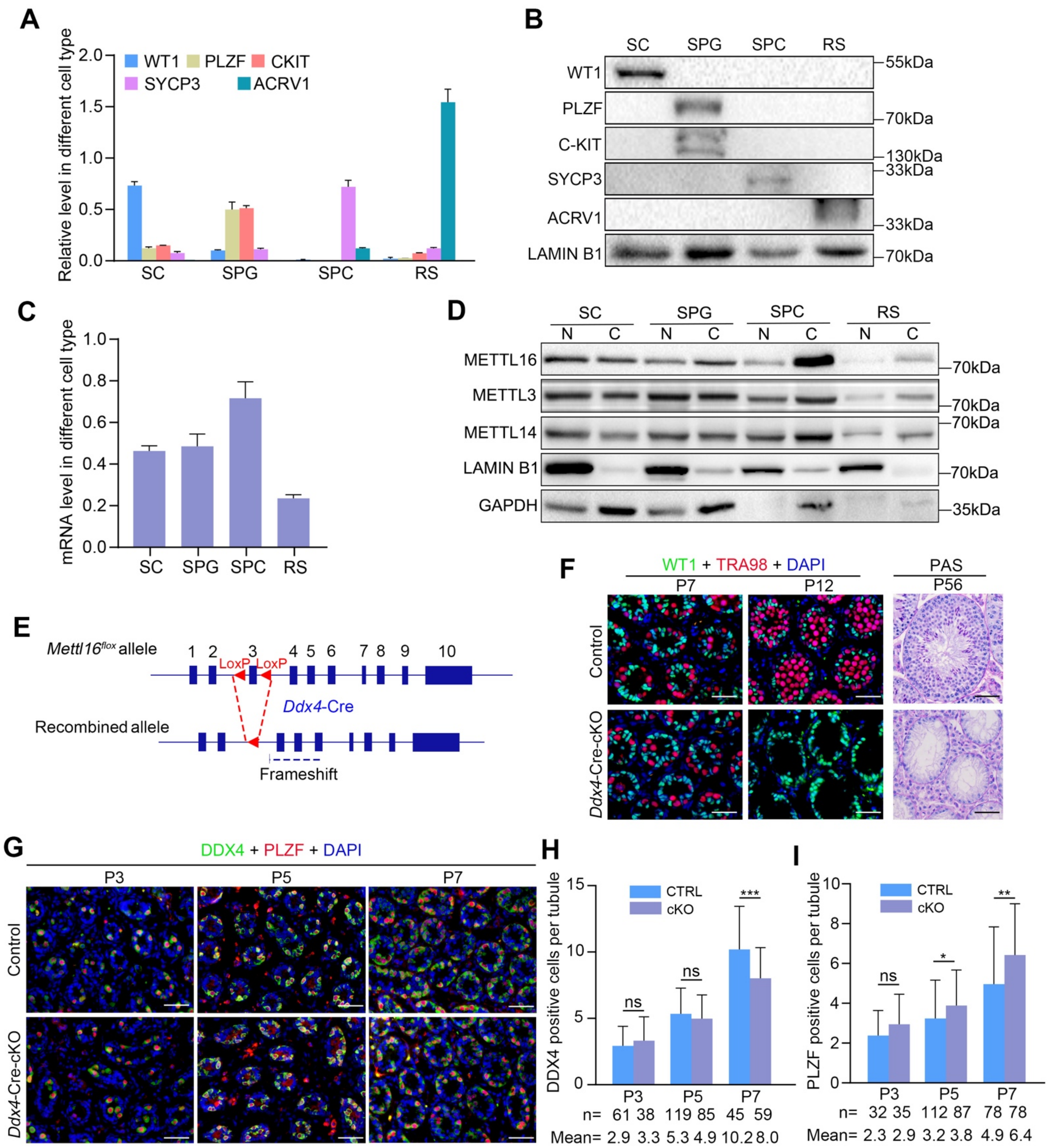

Figure S1. Expression pattern of *Mettl16* in testis and generation of conditional *Mettl16* knockout mice.

(**A-B**) qPCR and western blot assay with the markers WT1, PLZF, C-KIT, SYCP3 and ACRV1 to prove the purity of different types of isolated cells including spermatogonia (SPG), spermatocytes (SPC), round spermatids (RS), and Sertoli cells (SC). For western blot, LAMIN B1 serves as the loading control.

(**C**) qPCR analysis of the mRNA expression pattern of *Mettl16* in spermatogonia (SPG), spermatocytes (SPC), round spermatids (RS), and Sertoli cells (SC).

(**D**) Western blot assay of protein expression pattern of METTL16, METTL14, and METTL3 in nucleus (N) and cytoplasm (C) of spermatogonia (SPG), spermatocytes (SPC), round spermatids (RS), and Sertoli cells (SC), respectively. For western blot, LAMIN B1 and GAPDH serve as the loading controls.

(E) A schematic illustration of the strategy for conditional deletion of exon 3 of the *Mettl16* by *Ddx4-Cre* is shown.

(**F**) Immunofluorescence staining (*left* two panels) and Periodic acid-Schiff (PAS) staining (*right* panel) of testis sections from control and *Ddx4-Cre*; *Mettl16*$^{flox/-}$ mice at postnatal day 7 (P7), P12, and P56 (for PAS) are shown. WT1 stained for Sertoli cells and TRA98 stained for germ cells. DNA was stained with DAPI. Scale bars = 50 μm.

(**G**) Immunofluorescence staining of testis sections from control and *Ddx4-Cre*; *Mettl16*$^{flox/-}$ mice at postnatal day 3 (P3), P5 and P7 are shown. DDX4 stained for germ cells and PLZF stained for undifferentiated spermatogonia. DNA was stained with DAPI. Scale bars = 50 μm.

(**H-I**) Quantification of DDX4^{+} and PLZF^{+} cells per tubule for (G). The quantified data were presented as mean ± SEM. The indicated number of seminiferous tubules were counted from three Ctrl and cKO mice, respectively. ns, not significant. *$P < 0.05$, **$P < 0.01$, ***$P < 0.001$.

Figure S2 (Related to Figure 1)

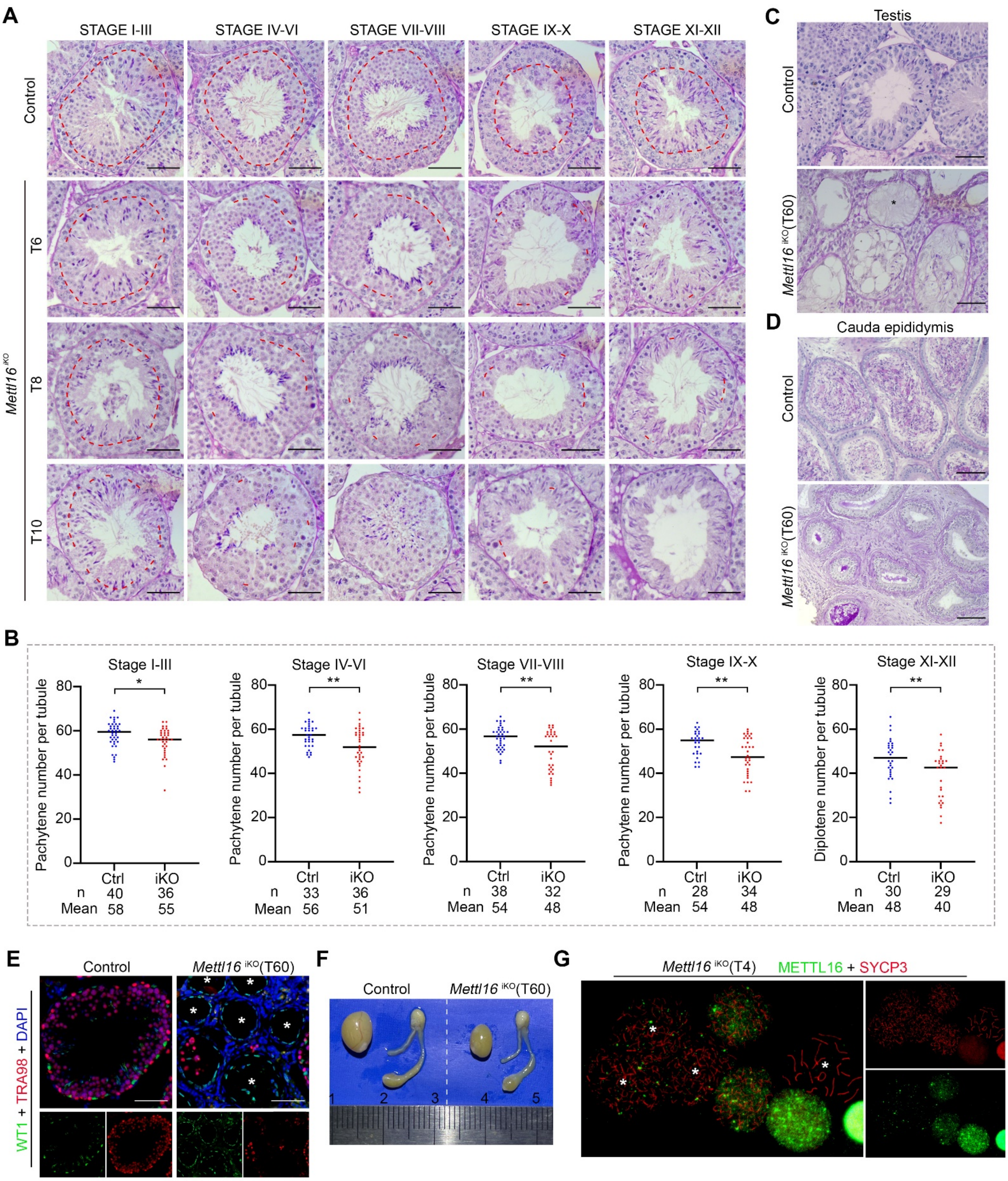

Figure S2. Histological and immunofluorescence staining analysis of *Mettl16*-deficient mouse testes.

(**A**) Histological analyses of different stages of testicular sections from Control and *Mettl16*^{iKO} adult mice at 6 dpt (T6), 8 dpt (T8), 10 dpt (T10) are shown. Twelve stages were divided into 5 groups: stage I-III, stage IV-VI, stage VII-VIII, stage IX-X, and stage XI-XII. The dashed red line indicates the layer of pachytene/diplotene spermatocytes. Scale bars = 50 μm.

(**B**) Quantification of pachytene or diplotene spermatocytes in stage I-III, stage IV-VI, stage VII-VIII, stage IX-X, and stage XI-XII seminiferous tubules of adult Control and *Mettl16*^{iKO} mice at T4 are shown. Data were presented as mean ± SEM. n = 5 mice. *P < 0.05, **P < 0.01.
(**C**) Histological analyses of seminiferous tubules in adult Control (Ctrl) and *Mettl16*^{iKO} (iKO) mice at T60 are shown. The asterisk labelled Sertoli-cell-only tubule. Scale bars = 50 μm.
(**D**) Histological analyses of cauda epididymides from adult Control and *Mettl16*^{iKO} mice at T60 are shown. Scale bars = 100 μm.
(**E**) Immunofluorescence staining of testis sections from control and *Ddx4-Cre*^{ERT2}; *Mettl16*$^{flox/-}$ mice at 60 dpt (T60) are shown. WT1 stained for Sertoli cells and TRA98 stained for germ cells. DNA was stained with DAPI. The asterisks labelled Sertoli-cell-only tubules. Scale bars = 50 μm.
(**F**) Gross morphology of the testes from adult Control and *Mettl16*^{iKO} mice at T60.
(**G**) Representative images of nuclear spread analysis showing METTL16 was undetectable in most spermatocytes.

Figure S3 (Related to Figure 2)

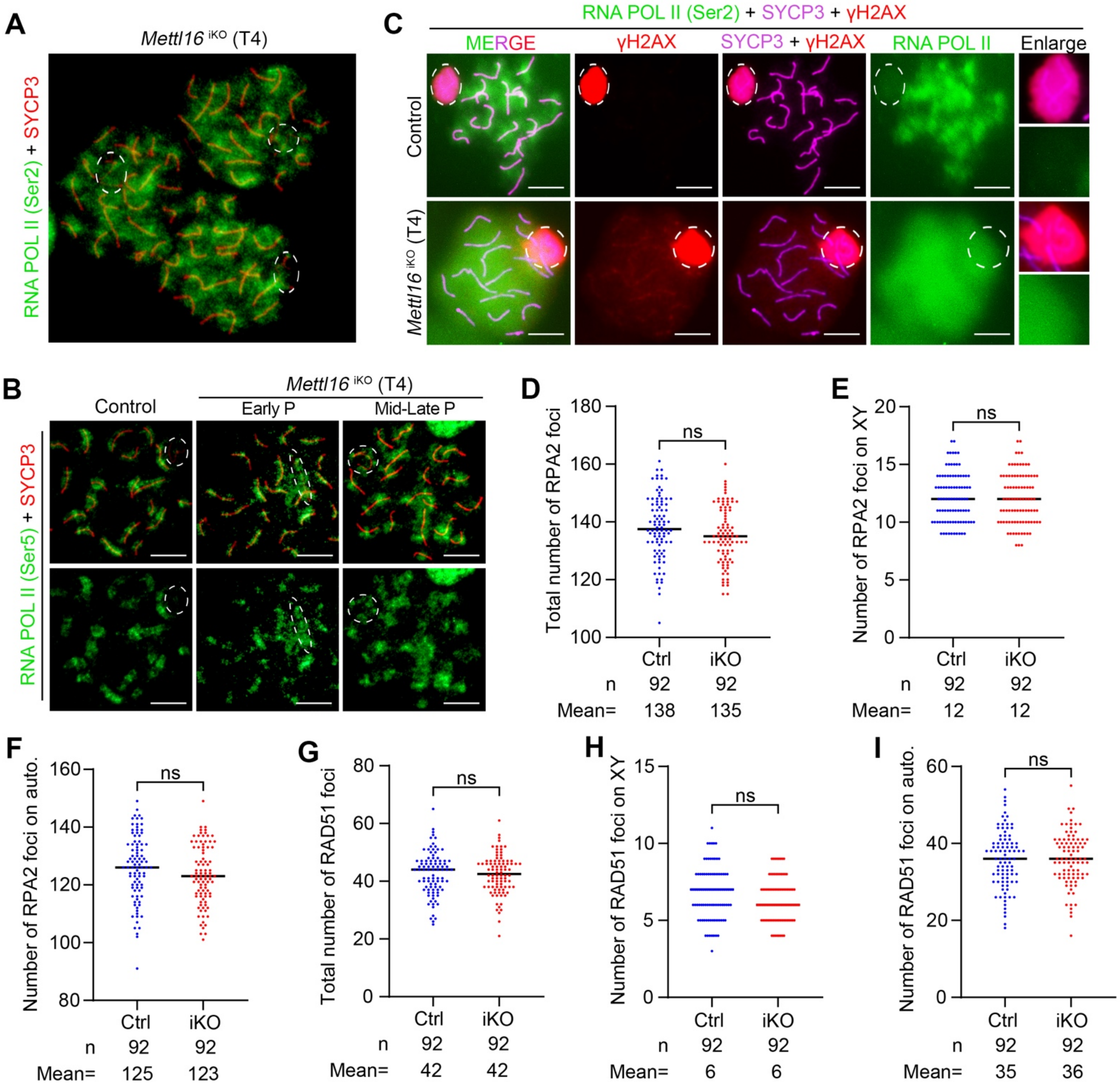

Figure S3. METTL16-depleted pachytene spermatocytes displayed abnormal RNA POL II signal but with normal homologous recombination.

(**A-C**) Representative images of nuclear spread analyses of RNA POL II with three different antibodies in pachytene spermatocytes from Control and *Mettl16*^{iKO} mice at T4 are shown. Abbreviation: Early P, early pachytene; Mid-late P, mid-late pachytene. White dashed lines indicate XY body. Scale bars = 10 μm.

(**D-F**) Qualification for RPA2 foci signal in whole early pachytene spermatocytes (D), and in XY chromosomes (E) or autosomes (F) of spermatocytes from Control (Ctrl) and *Mettl16*^{iKO} mice at T4. The indicated number of spermatocytes counted from three Ctrl and iKO mice are shown at the bottom of the histogram, respectively. The quantified data were presented as mean ± SEM. ns, not significant. Abbreviation: Auto., autosomes.

(**G-I**) Qualification for RAD51 foci signal in whole early pachytene spermatocytes (G), and in XY

chromosomes (H) or autosomes (I) of spermatocytes from Control (Ctrl) and *Mettl16*^{iKO} mice at T4. The indicated number of spermatocytes counted from three Ctrl and iKO mice are shown at the bottom of the histogram, respectively. The quantified data were presented as mean ± SEM. ns, not significant. Abbreviation: Auto., autosomes.

Figure S4 (Related to Figure 2)

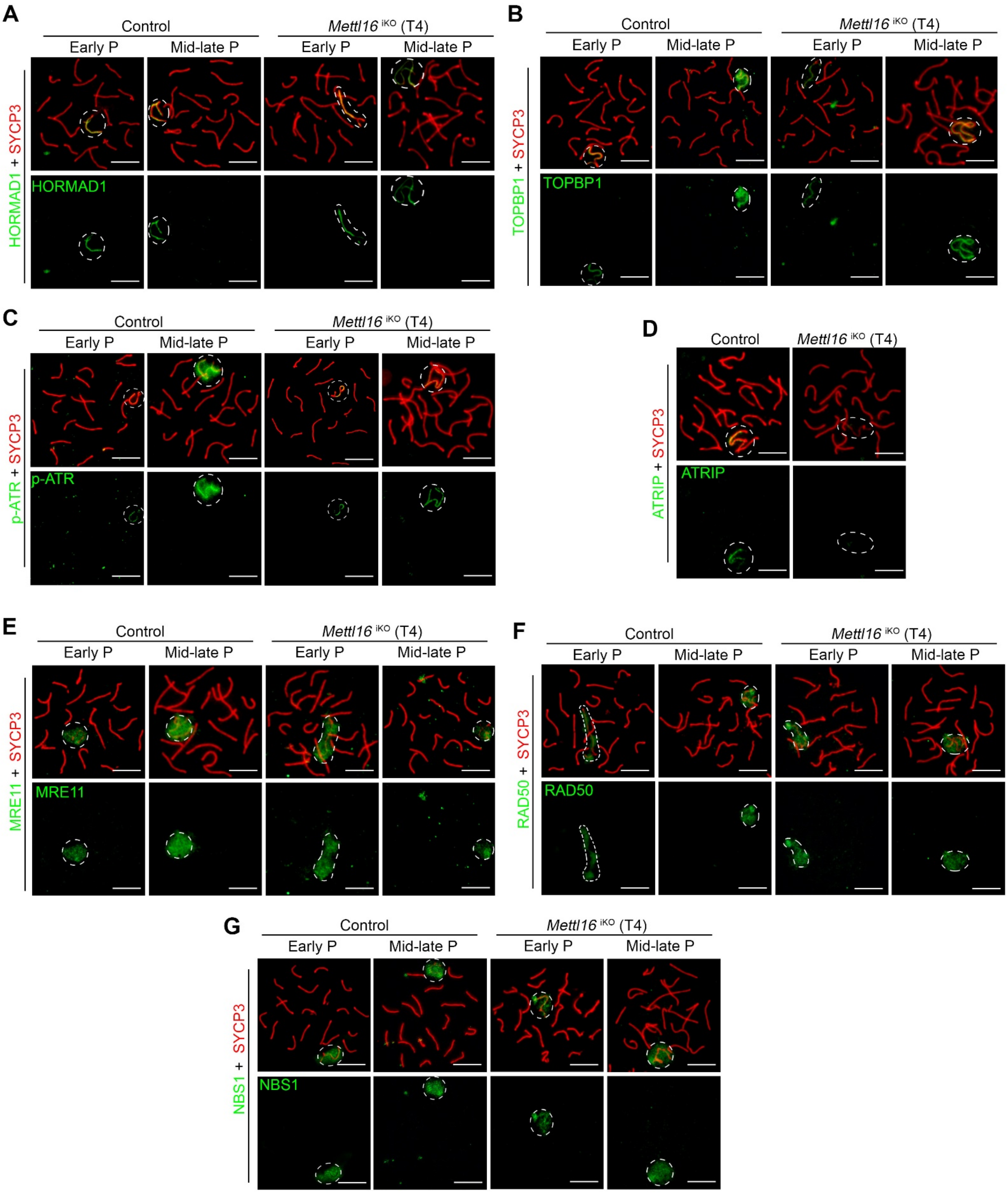

Figure S4. METTL16 is involved in the amplification of DDR factors but has no effect on MRN complex localization.

(**A-D**) Representative images of nuclear spread analysis of HORMAD1 (A), TOPBP1 (B), p-ATR (C), ATRIP (D) in pachytene spermatocytes from Control and *Mettl16*^{iKO} mice at T4 are shown. Abbreviation: Early P, early pachytene; Mid-late P, mid-late pachytene. White dashed lines indicate XY body. Scale bars = 10 μm.

(**E-G**) Nuclear spread analyses of MRN complex [MRE11 (E)-RAD50 (F)-NBS1 (G)] in Control and *Mettl16*^{iKO} mouse pachytene spermatocytes at T4 are shown. Abbreviation: Early P, early pachytene; Mid-late P, mid-late pachytene. White dashed lines indicate XY body. Scale bars = 10 μm.

Figure S5 (Related to Figure 3)

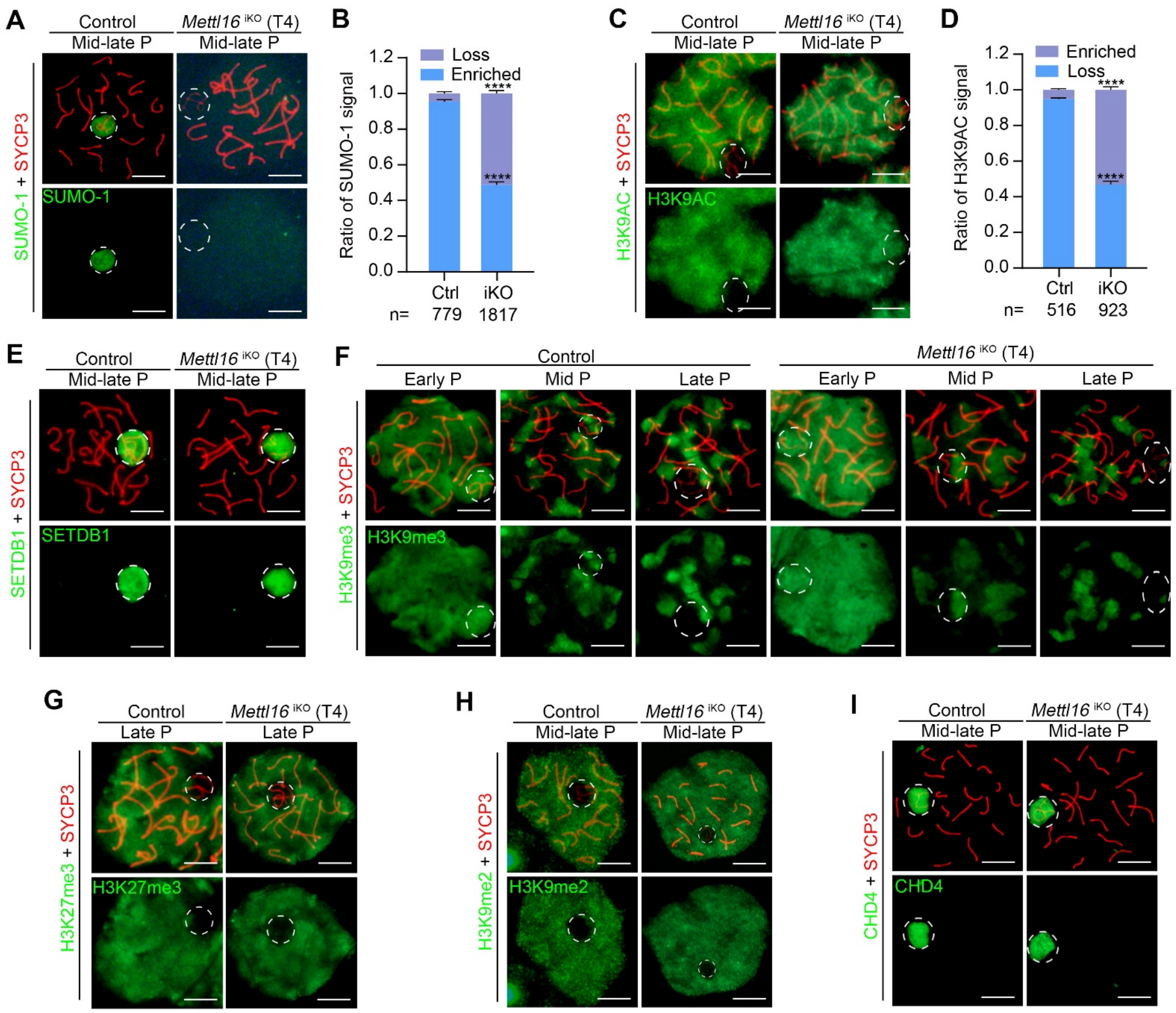

Figure S5. METTL16 affects SUMO-1 and H3K9AC instead of SETDB1-H3K9me3, H3K27me3, H3K9me2, and CHD4 in pachytene spermatocytes.

(**A-B**) Representative images of nuclear spread analysis and quantification of signals for SUMO-1 in mid-late pachytene (Mid-late P) spermatocytes from Control and *Mettl16*^{iKO} mice at T4 are shown. The abbreviation in quantification part: Loss, no signal in XY body; Enriched, signal in XY body. The quantified data were presented as mean ± SEM. ****$P < 0.0001$. Scale bars = 10 μm.

(**C-D**) Representative images of nuclear spread analysis and quantification of signals for H3K9AC in mid-late pachytene (Mid-late P) spermatocytes from Control and *Mettl16*^{iKO} mice at T4 are shown. The abbreviation in quantification part: Loss, no signal in XY body; Enriched, signal in XY body. The quantified data were presented as mean ± SEM. ****$P < 0.0001$. Scale bars = 10 μm.

(**E**) Representative images of nuclear spread analysis of signals for SETDB1 in mid-late pachytene (Mid-late P) spermatocytes from Control and *Mettl16*^{iKO} mice at T4 are shown. Scale bars = 10 μm.

(**F**) Representative images of nuclear spread analysis of signals for H3K9me3 in pachytene spermatocytes from Control and *Mettl16*^{iKO} mice at T4 are shown. The abbreviation in images: Early P, early pachytene; Mid P, mid pachytene; Late P, late pachytene. Scale bars = 10 μm.

(**G**) Representative images of nuclear spread analysis of signals for H3K27me3 in late pachytene

(Late P) spermatocytes from Control and *Mettl16*^{iKO} mice at T4 are shown. Scale bars = 10 µm.
(**H**) Representative images of nuclear spread analysis of signals for H3K9me2 in mid-late pachytene (Mid-late P) spermatocytes from Control and *Mettl16*^{iKO} mice at T4 are shown. Scale bars = 10 µm.
(**I**) Representative images of nuclear spread analysis of signals for CHD4 in mid-late pachytene (Mid-late P) spermatocytes from Control and *Mettl16*^{iKO} mice at T4 are shown. Scale bars = 10 µm.

Figure S6 (Related to Figures 4-5)

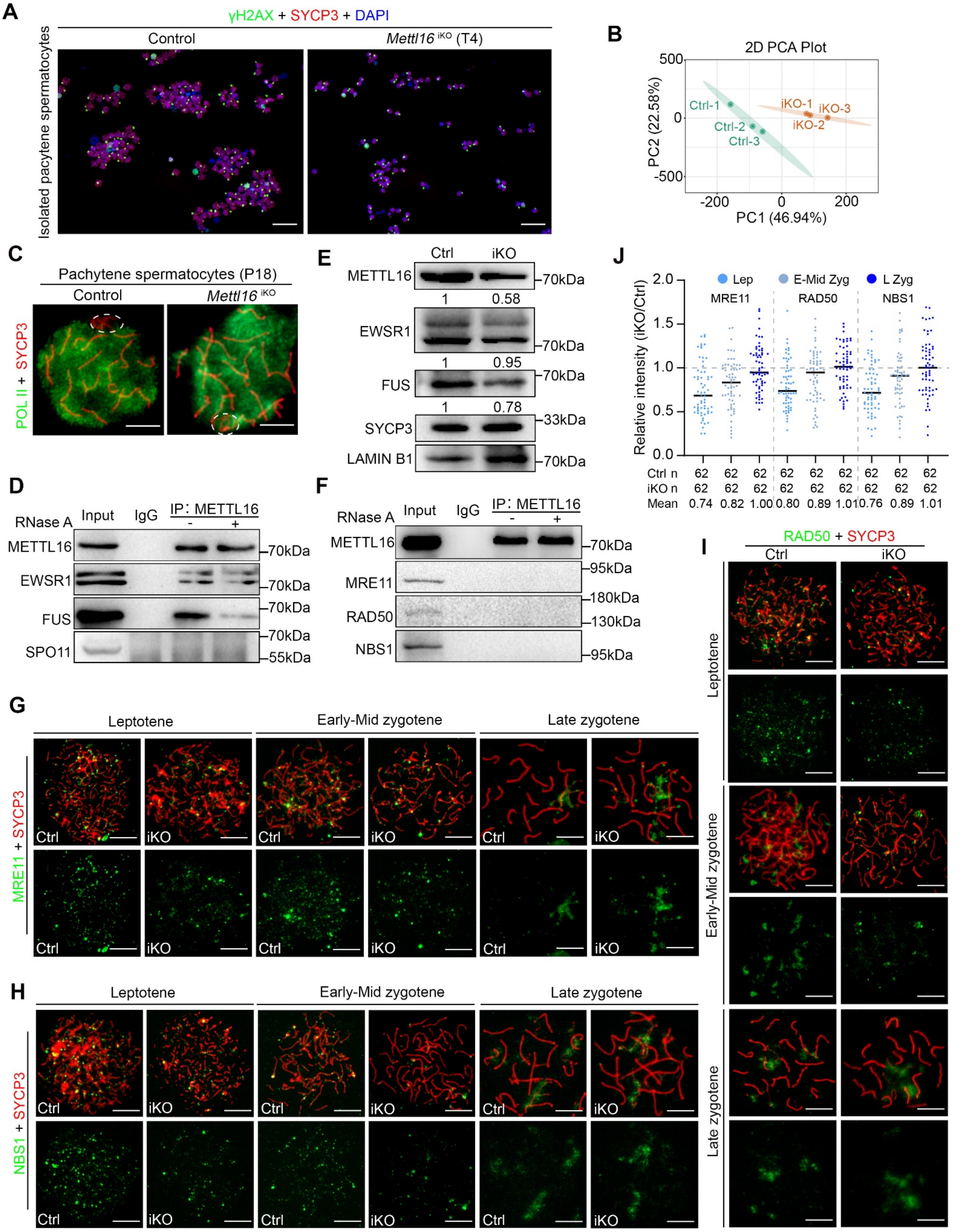

Figure S6. METTL16 affects transcriptome of pachytene spermatocytes and DSB formation in the first wave of spermatogenesis.

(**A**) Immunofluorescent staining analysis of the purity of pachytene spermatocytes isolated from Control (Ctrl) and *Mettl16*^{iKO} mice at T4 using STA-PUT method. Red, SYCP3. Green, γH2AX. Scale bars = 100 μm.
(**B**) Principal components analysis (PCA) of Control (Ctrl) and *Mettl16*^{iKO} (iKO) pachytene spermatocytes. Green region indicates the Ctrl group, and orange region indicates the iKO group, respectively. n = 3 mice.
(**C**) Representative images of nuclear spread analysis of POL II in pachytene spermatocytes from Control and *Mettl16*^{iKO} mice at P18. White dashed lines indicate XY body. Scale bars = 10 μm.
(**D**) Co-Immunoprecipitation (Co-IP) assay to validate the interaction between METTL16 and DSB-related proteins FUS/EWSR1 using METTL16 antibody with or without RNase A.
(**E**) Western blot and quantification of FUS/EWSR1 in testes of Ctrl and iKO mice at P10. LAMIN B1 represents total protein, while SYCP3 was used as a normalized control. n = 8 mice.
(**F**) Co-Immunoprecipitation (Co-IP) assay to validate the interaction between METTL16 and MRN complex using METTL16 antibody with or without RNase A.
(**G-I**) Representative images of nuclear spread analysis of the MRN complex-MRE11 (G), RAD50 (H), and NBS1 (I) in leptotene, early-mid zygotene, and late zygotene spermatocytes from juvenile Ctrl and iKO male mice are shown. Scale bars = 10 μm.
(**J**) Quantification of MRE11, RAD50 and NBS1 signal (iKO/Ctrl) in different type of spermatocytes are shown. The indicated number of spermatocytes counted from three Ctrl and iKO mice are shown at the bottom of the plots, respectively. Abbreviation: Lep, leptotene; E-Mid Zyg, early-mid zygotene; L Zyg, late zygotene.

Figure S7 (Related to Figure 6)

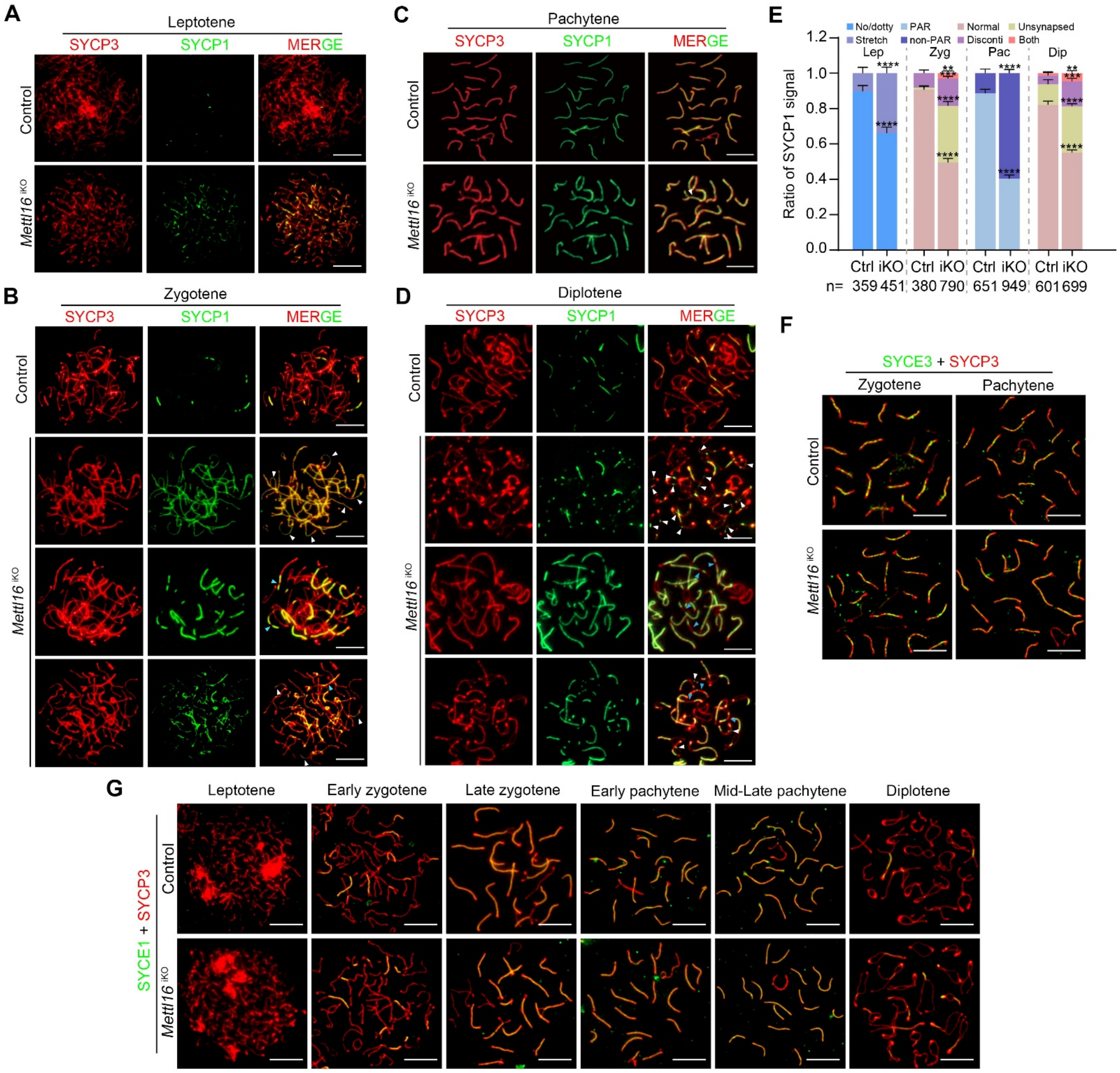

Figure S7. METTL16 affects SYCP1 localization during the first wave of spermatogenesis.
(**A-D**) Representative images of nuclear spread analysis of SYCP1 in leptotene (A), zygotene (B), pachytene (C) and diplotene (D) spermatocytes from juvenile Control and *Mettl16*^{iKO} mice. White arrowheads indicate SYCP1 localization on unsynapsed chromosomes. Blue arrowheads indicate discontinuous SYCP1 localization on synapsed homologous chromosomes. Scale bars = 10 μm.
(**E**) Quantification of different types of SYCP1 signal in spermatocytes for (A-D). Abbreviation: No/dotty, no/dotty SYCP1 signal; Stretch, short stretches of SYCP1 signal; PAR, SYCP1 only on the PAR; non-PAR, SYCP1 on XY axes beyond PAR; Normal, normal SYCP1 localization; Disconti, discontinuous SYCP1 on synapsed homologous chromosomes; Unsynapsed, SYCP1 on unsynapsed chromosomes; Both, cells containing both unsynapsed and disconti; Lep, leptotene; Zyg, zygotene; Pac, pachytene; Dip, diplotene. The quantified data were presented as mean ± SEM. n = 3 mice. **P < 0.01, ***P < 0.001, ****P < 0.0001.
(**F-G**) Representative images of nuclear spread analysis of SYCE1 (G) and SYCE3 (F) in spermatocytes from Control and *Mettl16*^{iKO} juvenile mice. Scale bars = 10 μm.

Figure S8 (Related to Figure 7)

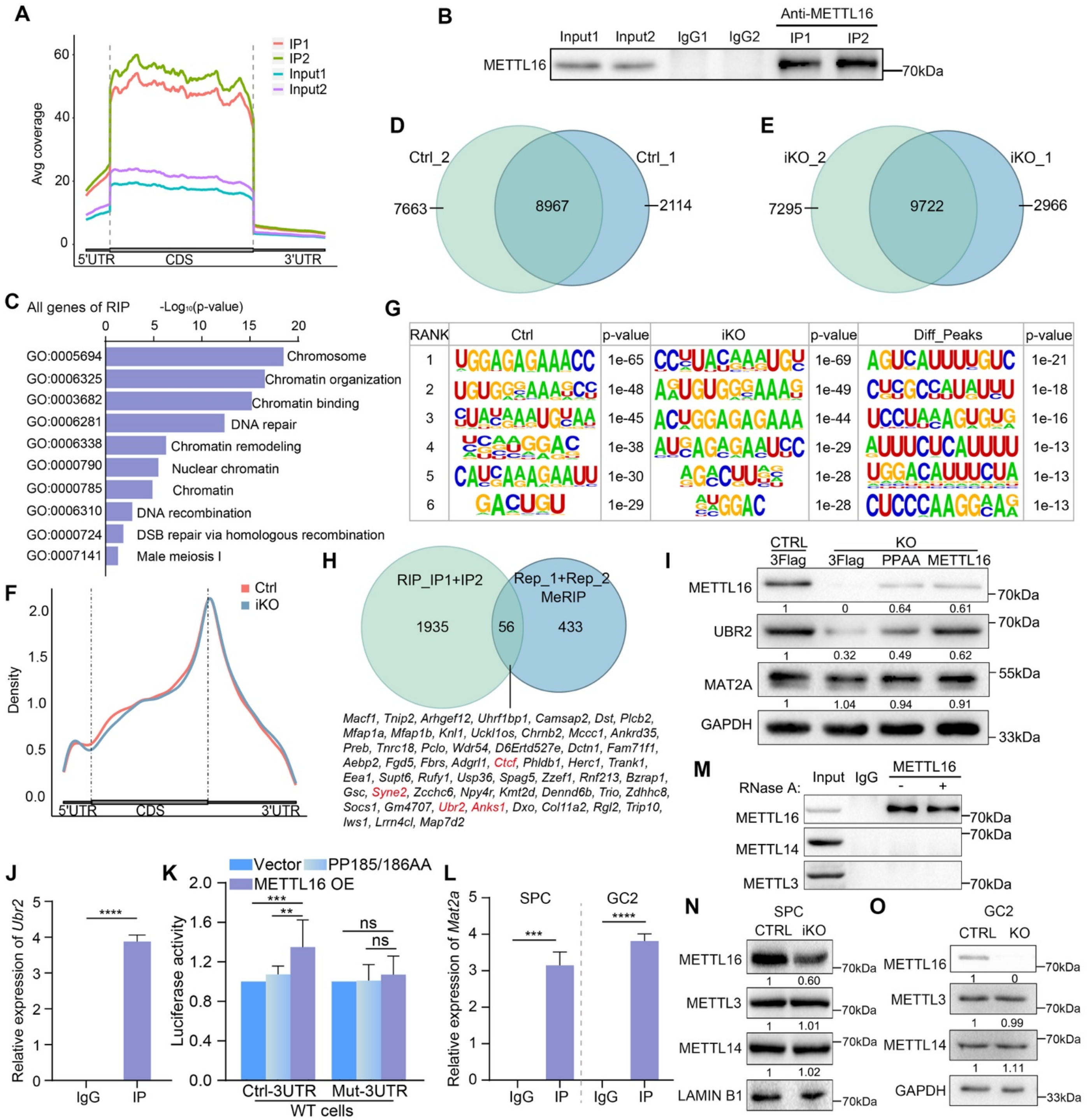

Figure S8. The m^{6}A landscape of spermatocytes upon *Mettl16* depletion and characteristics of METTL16 targeted genes.

(**A**) The distribution of METTL16 binding sites in the RNAs of isolated spermatocytes identified by RIP-seq (RIP-sequencing).

(**B**) Immunoprecipitation (Co-IP) assay to validate the effectiveness of RIP assay using METTL16 antibody.

(**C**) Gene ontology (GO) analysis of METTL16 targeted genes (all genes identified by RIP-Seq).

(**D-E**) Venn diagram showing the overlaps of m^{6}A peaks in replicate 1 and 2 in control (D) and iKO (E) respectively identified by MeRIP-seq (MeRIP-sequencing).

(**F**) The m^{6}A-peak density in control and *Mettl16*^{iKO} spermatocytes.

(**G**) The top six enriched m^{6}A-specific motifs from Ctrl, iKO and differential peaks are shown along

with *P* value analyzed by HOMER.

(**H**) Venn diagram showing the overlap of METTL16 targeted genes and genes with decreased m^6A modification. The red genes are highly associated with male meiosis.

(**I**) Western blot and quantification for METTL16, UBR2, and MAT2A in Ctrl or *Mettl16* KO GC2 cells with exogeneous overexpression of CMV-3xFlag, 3xFlag-METTL16-PP185/186AA (PPAA), and 3xFlag-METTL16. GAPDH serves as loading control.

(**J**) RIP-qPCR to confirm *Ubr2* as a target of METTL16 in GC2 cells. n = 3 mice. ****$P < 0.0001$.(**K**) Luciferase activities of control (Ctrl) and Mut 3'UTR vectors with exogeneous overexpression of CMV-3xFlag (Vector), 3xFlag-METTL16-PP185/186AA, and 3xFlag-METTL16 in WT GC2 cells. Abbreviation: OE, overexpression. ***$P < 0.001$, **$P < 0.01$. ns, not significant.

(**L**) RIP-qPCR to confirm *Mat2a* as a target of METTL16 in spermatocytes (SPC) and GC2 cells. n = 3 mice. ***$P < 0.001$, ****$P < 0.0001$.

(**M**) Immunoprecipitation (Co-IP) assay to validate the interaction between METTL16, METTL14 and METTL3 in spermatocytes using METTL16 antibody with or without RNase A.

(**N**) Western blot analysis and quantification of METTL16, METTL14 and METTL3 in isolated spermatocytes (SPC) from Control (Ctrl) and *Mettl16*^{iKO} (iKO) juvenile mice. LAMIN B1 serves as loading control.

(**O**) Western blot analysis and quantification of METTL16, METTL14 and METTL3 in Ctrl and *Mettl16* KO GC2 cells. GAPDH serves as loading control.

Figure S9 (Related to Figure 8)

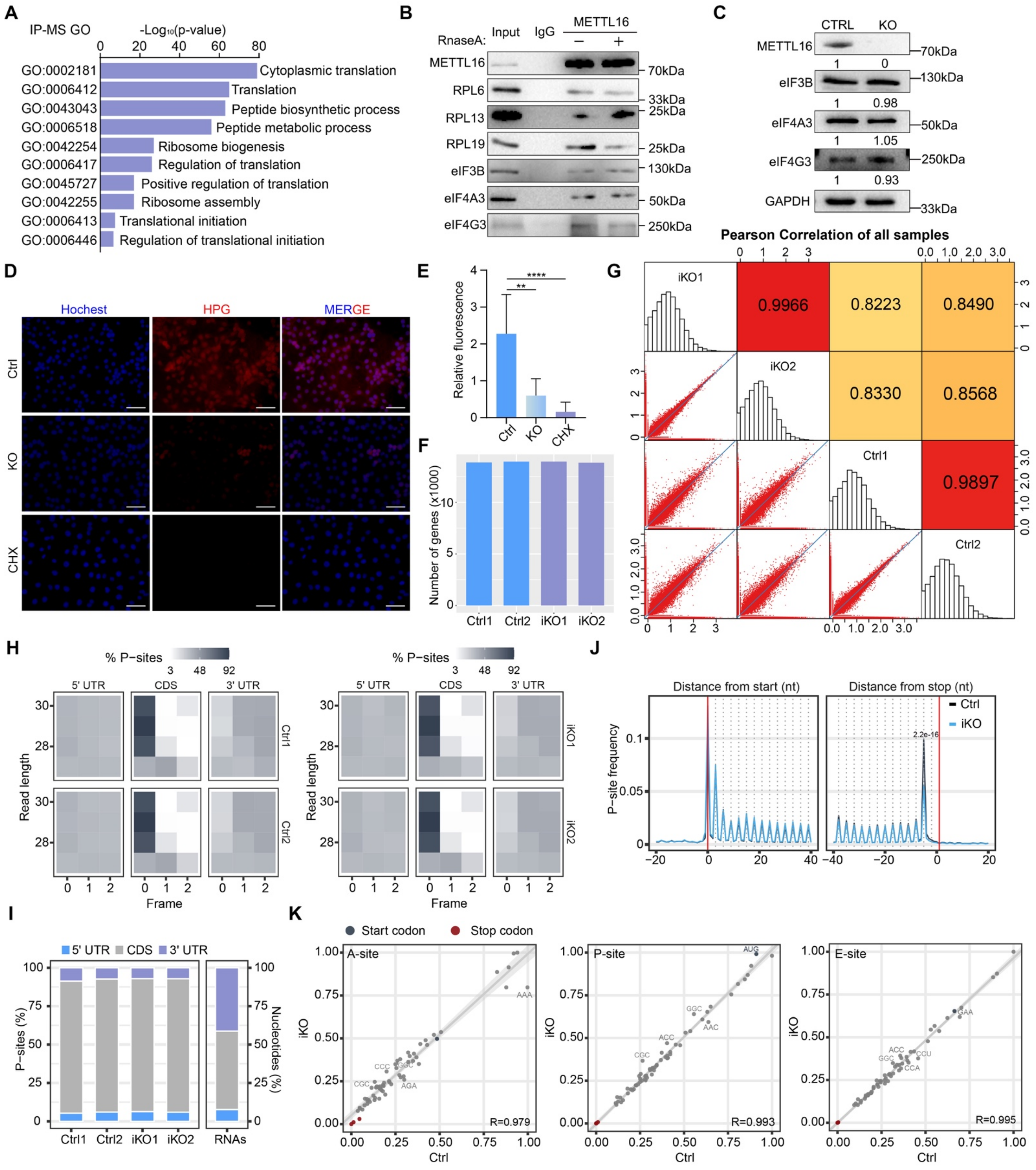

Figure S9. METTL16 affects translation process in spermatocytes and GC2 cells.

(**A**) GO analysis of the potential interactors of METTL16 identified by IP-MS.

(**B**) Co-Immunoprecipitation (Co-IP) assay to validate the interactions between METTL16, RPL6, RPL13, RPL19, eIF3B, eIF4A3, and eIF4G3 using METTL16 antibody with or without RNase A in GC2 cells.

(**C**) The protein levels and quantification of eIF3B, eIF4A3, and eIF4G3 in Ctrl and *Mettl16* KO GC2

cells. GAPDH serves as loading control.
(**D**) Immunofluorescence analyses of *Mettl16* KO GC2 cells and control cells treated or not treated with cycloheximide (CHX). Nascent protein synthesis was detected by staining with HPG-594 (red) and DNA was stained using Hochest (blue). Scale bars = 50 μm.
(**E**) Quantification of fluorescent intensity of HPG/ Hochest between Ctrl, *Mettl16* KO, and CHX GC2 cells. $^{**}P < 0.01$, $^{****}P < 0.0001$.
(**F**) Bar plots showing the numbers of detected genes (TPM > 1) by the Ribo-seq data (two replicates).
(**G**) Scatter plots comparing the Ribo-seq data of two replicates of Ctrl and iKO spermatocytes. Spearman correlations are shown.
(**H**) Heatmap of the percentages of footprints matching the reading frames in Ctrl and iKO spermatocytes.
(**I**) Bar plots showing the mapping reads distribution in 5UTR, CDS, and 3UTR regions in Ctrl and iKO spermatocytes.
(**J**) Overlay meta-profiles for mRNAs enriched in Ctrl and iKO spermatocytes.
(**K**) Comparison of codon usage index at P/A/E-site between Ctrl and iKO spermatocytes.
